# Supplementary material for: Unzipped chromosome-level genomes reveal allopolyploid nematode origin pattern as unreduced gamete hybridization
Source: Nat Commun. 2023 Nov 7;14:7156. doi: 10.1038/s41467-023-42700-w (PMC10630426; doi:10.1038/s41467-023-42700-w)
Supplement: Supplementary file 1 — Supplementary Information [file 41467_2023_42700_MOESM1_ESM.pdf]

Supplemental information for "Unzipped chromosome-level genomes reveal allopolyploid nematode origin pattern as unreduced gamete hybridization"

Dadong Dai[1, 2]†, Chuanshuai Xie[1, 2]†, Yayi Zhou[1, 3], Dexin Bo[1, 3], Shurong Zhang[1, 2], Shengqiang Mao[1, 3], Yucheng Liao[1, 3], Simeng Cui[1, 2], Zhaolu Zhu[1, 3], Xueyu Wang[1, 2], Fanling Li[1,2], Donghai Peng[1, 2]*, Jinshui Zheng[1, 3]*, and Ming Sun[1, 2]*

[1]State Key Laboratory of Agricultural Microbiology, Hubei Hongshan Laboratory, Huazhong Agricultural University, Wuhan, 430070, China.

[2]College of Life Science and Technology, Huazhong Agricultural University, Wuhan, 430070, China.

[3]Hubei Key Laboratory of Agricultural Bioinformatics, College of Informatics, Huazhong Agricultural University, Wuhan, 430070, China.

*Corresponding author Email:

m98sun@mail.hzau.edu.cn, jszheng@mail.hzau.edu.cn,

donghaipeng@mail.hzau.edu.cn

†These authors contributed equally to this work.

Supplementary Figures

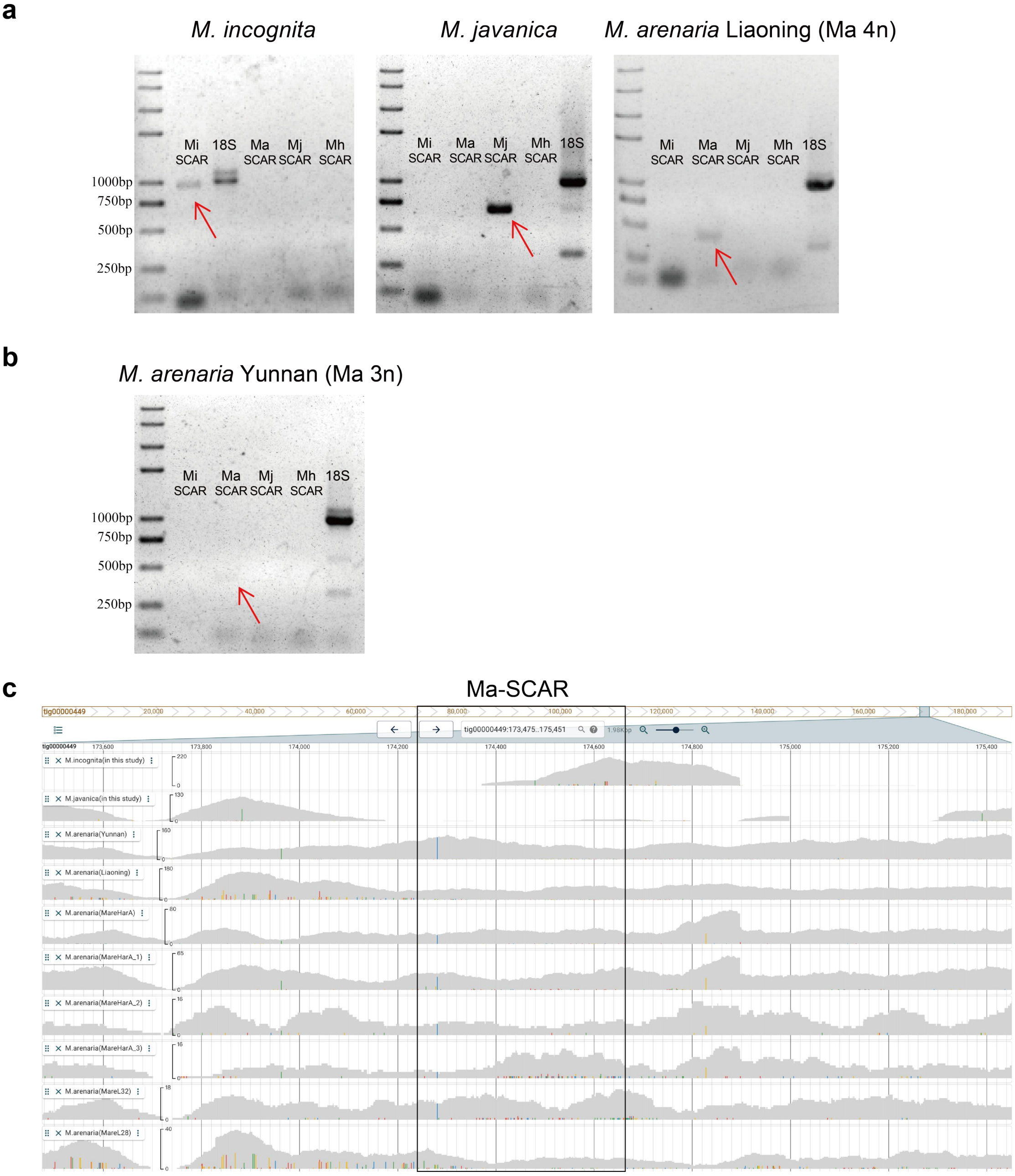

Supplementary Figure 1 Species identification of four polyploid *Meloidogyne* species. (**a**) Identification of root-knot nematode species by SCAR characteristic primers. (**b**) The left picture is the SCAR identification of *M. arenaria* Yunnan in the early stage of this study, only a weak band was observed with the Ma SCAR marker initially. The primers used for each track are marked in the figure (**a**) and (**b**), and the templates of all tracks are the same template, so as to test which specific primers can amplify the bands of the sample, and to determine the species. The red arrows show the corresponding PCR bands. (**c**) Read coverage in Ma-SCAR target sequence. Reference sequences are published Ma genome (GCA_003133805.1). Ma-SCAR target region are located at tig00000449:174235-174642. Reads of *M. arenaria* Yunnan completely cover the Ma-SCAR target region, which prove that the nematode sample from Yunnan are *M. arenaria.* The sequence alignment is visualized using JBrowse2. Source data are provided as a Source Data file.

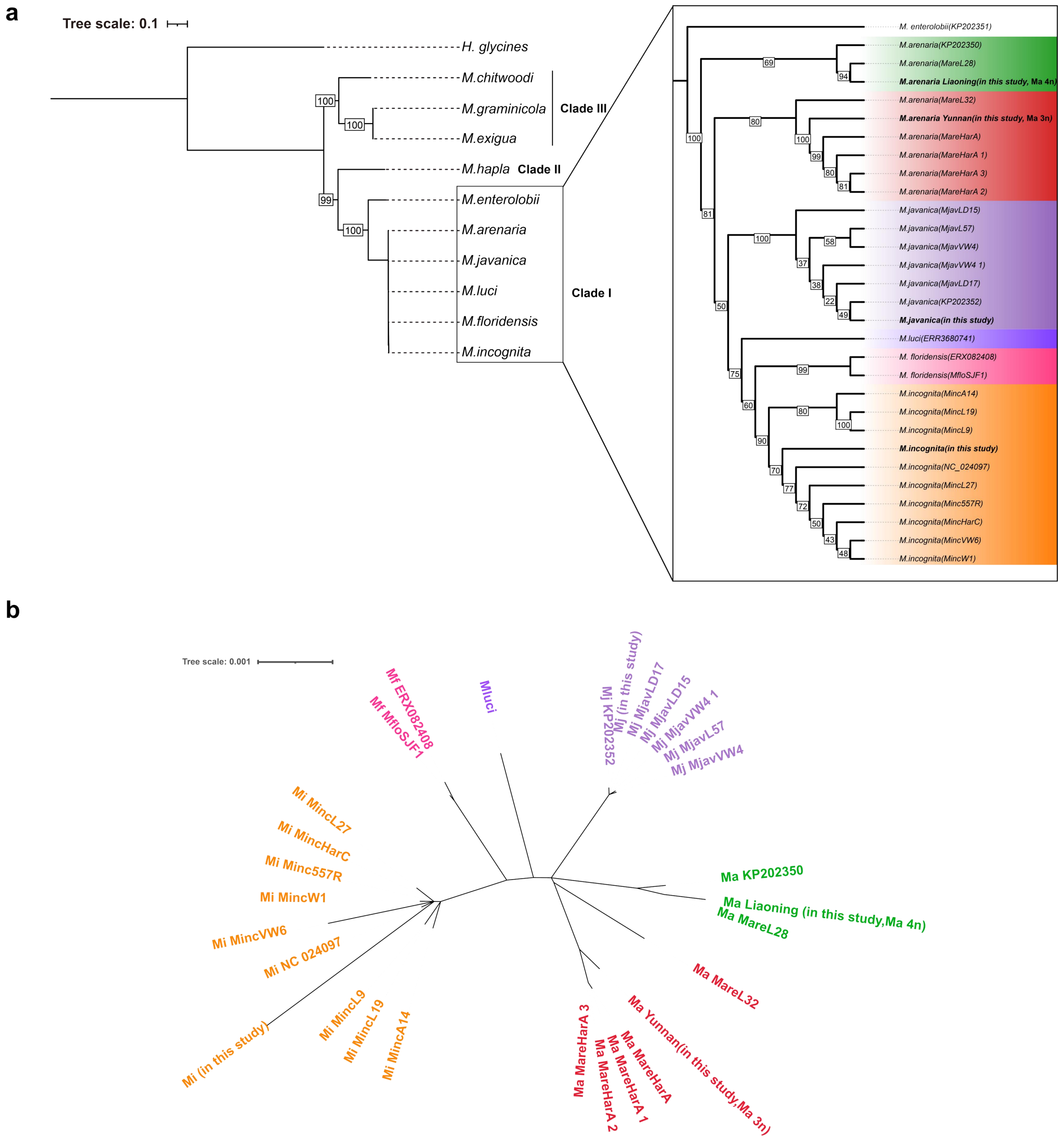

Supplementary Figure 2 Mitochondrial phylogeny for *Meloidogyne* species. (a) Mitochondrial tree for all *Meloidogyne* species. The maximum-likelihood tree is constructed from concatenated supermatrix of all mitochondrial protein coding genes. Bootstrap are shown and Clade are defined by Tandingan De Ley *et al*. Species are marked in different colours. Ma has two branches, the branch where Ma Yunnan is located and the branch where other Ma Liaoning is located are marked in red and green. (**b**) Unrooted mitochondrial tree for Clade I RKNs. The maximum-likelihood tree is constructed from concatenated supermatrix of all mitochondrial protein coding genes and rRNA genes. Two Ma branches and Mj form a trifurcating structure. *M. enterolobii* is excluded from unrooted tree.

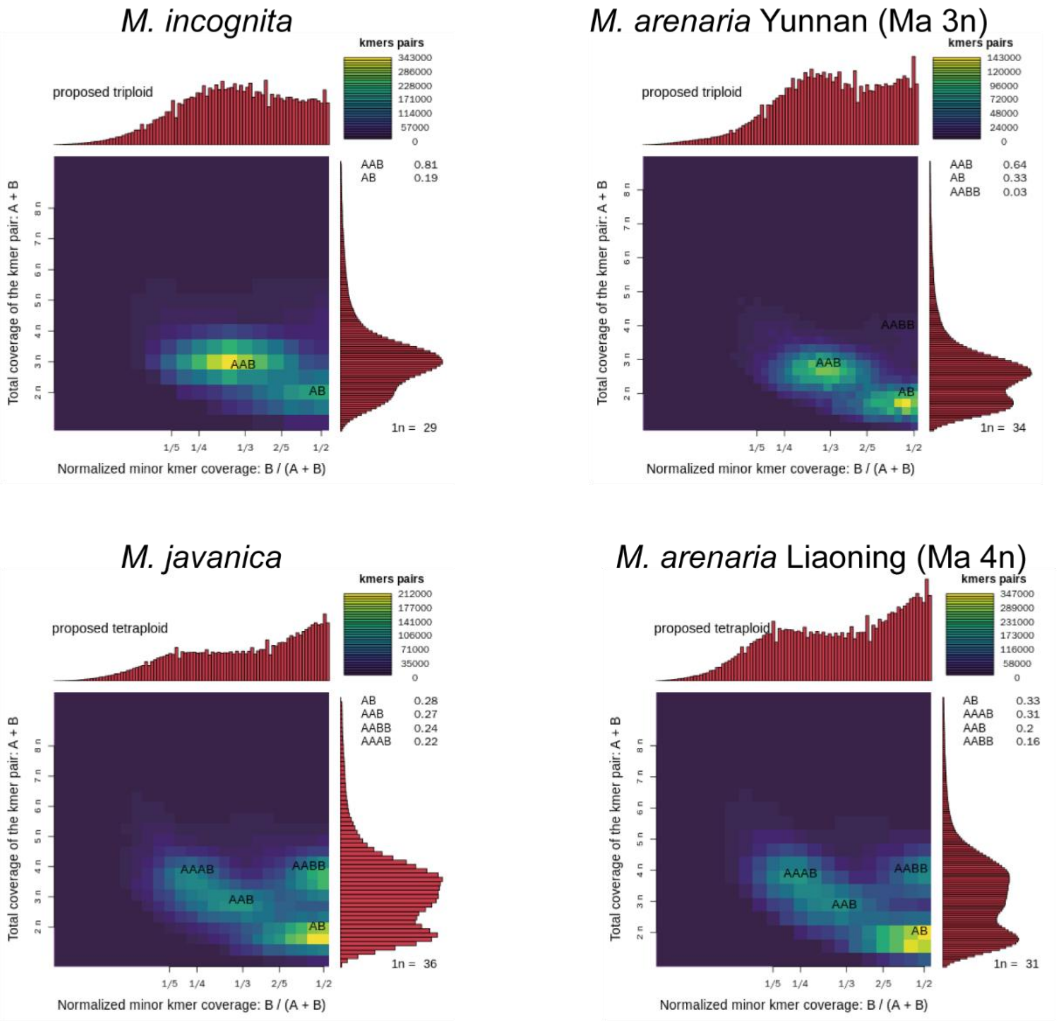

Supplementary Figure 3 Ploidy estimation of four polyploid *Meloidogyne* species. Corrected PacBio long reads are used to Smudgeplot analysis.

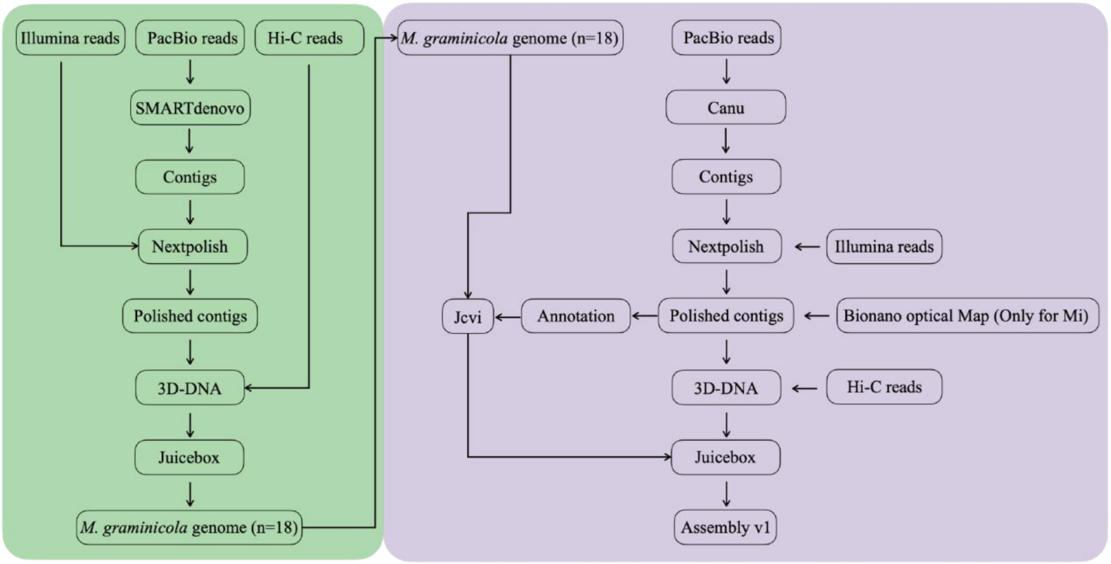

Supplementary Figure 4 Workflow for construction of assembly v1.

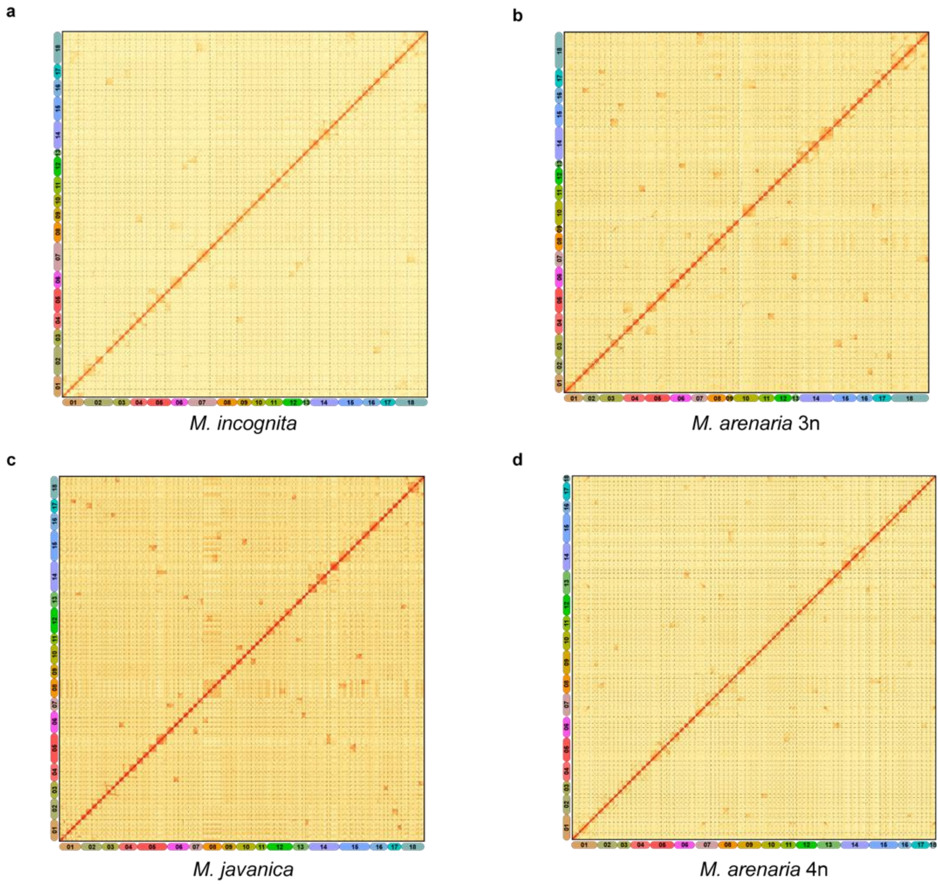

Supplementary Figure 5 Genome-wide Hi-C heatmap of polyploid *Meloidogyne* species. (**a**) The whole genome Hi-C heatmap of *M. incognita*, numbers 1-18 on the X-axis and Y-axis represent chromosomes 1-18 of *M. graminicola*. (**b**) The whole genome Hi-C heatmap of *M. arenaria* 3n, numbers 1-18 on the X-axis and Y-axis represent chromosomes 1-18 of *M. graminicola*. (**c**) The whole genome Hi-C heatmap of *M. javanica*, numbers 1-18 on the X-axis and Y-axis represent chromosomes 1-18 of *M. graminicola*. (**d**) The whole genome Hi-C heatmap of *M. arenaria* 4n, numbers 1-18 on the X-axis and Y-axis represent chromosomes 1-18 of *M. graminicola*. The color represents the contact signal between two 150kb windows.

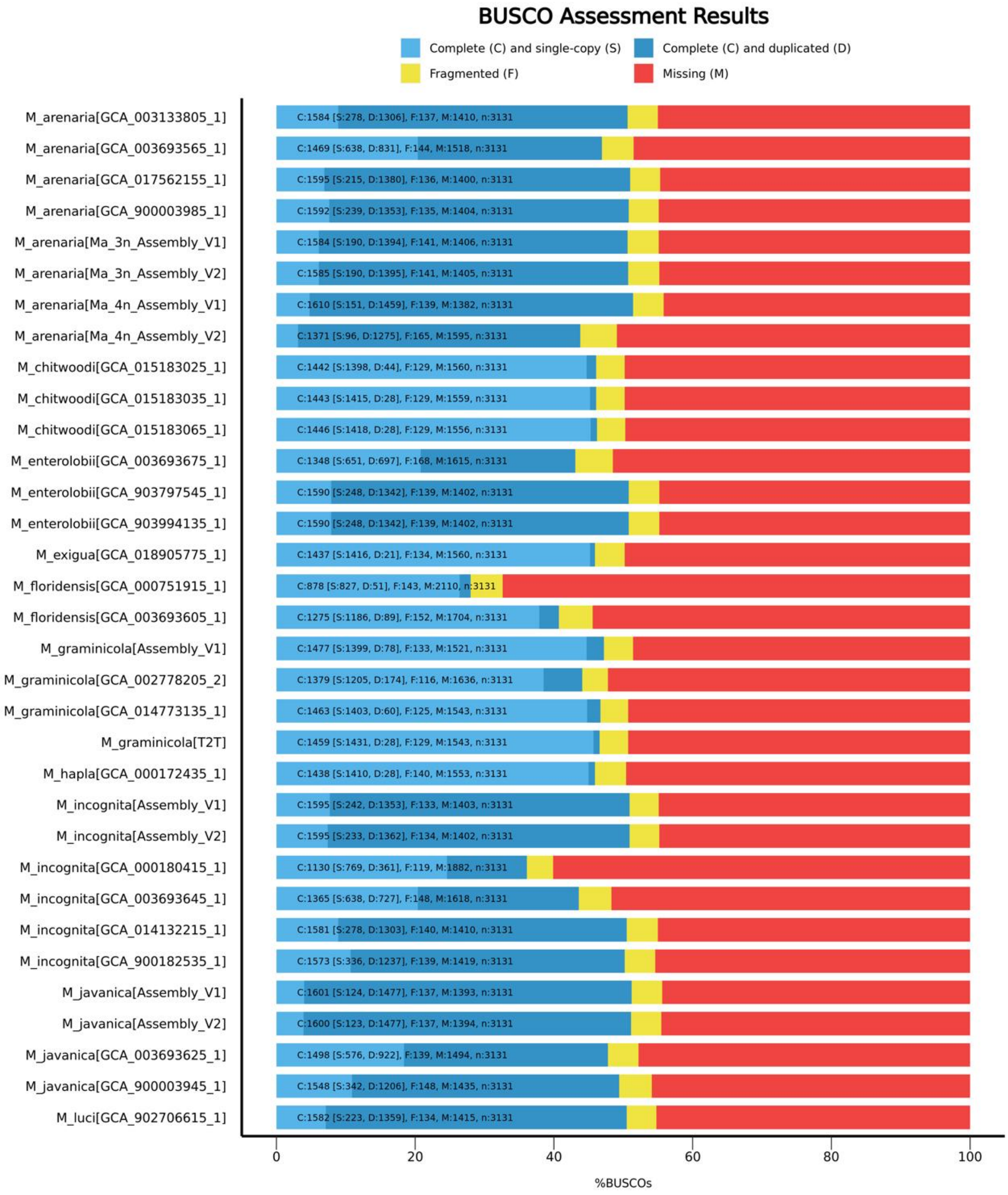

Supplementary Figure 6 BUSCO assessment for genomes. Genomes include assembly in this study and other *Meloidogyne* species genome available in NCBI. Source data are provided as a Source Data file.

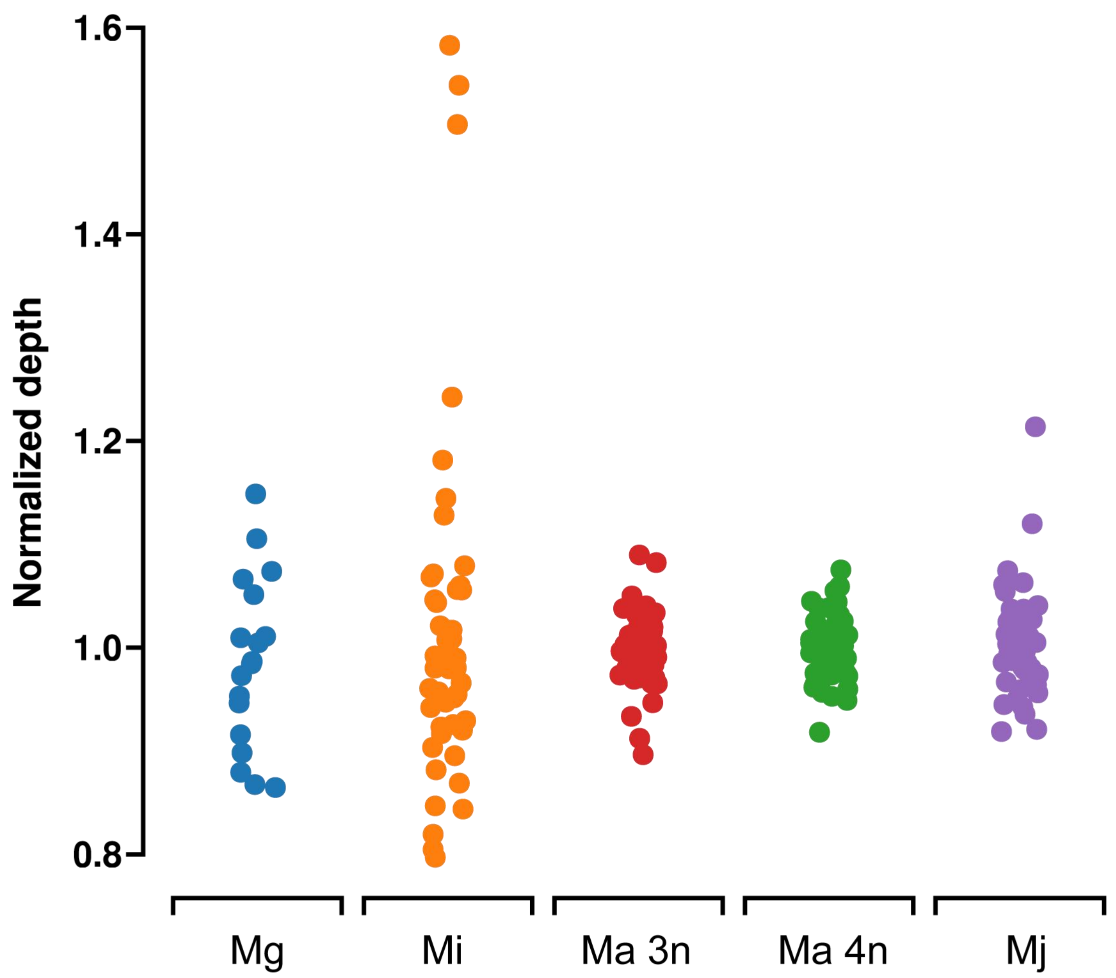

Supplementary Figure 7 Normalized sequencing depth of scaffolds belonging to five *Meloidogyne* species. Each dot represents the median value of normalized depth for one scaffold in corresponding species. The sequencing depth of most scaffolds is uniform, except for three scaffolds in Mi, indicating that the three scaffolds partially collapsed during genome assembly. Source data are provided as a Source Data file.

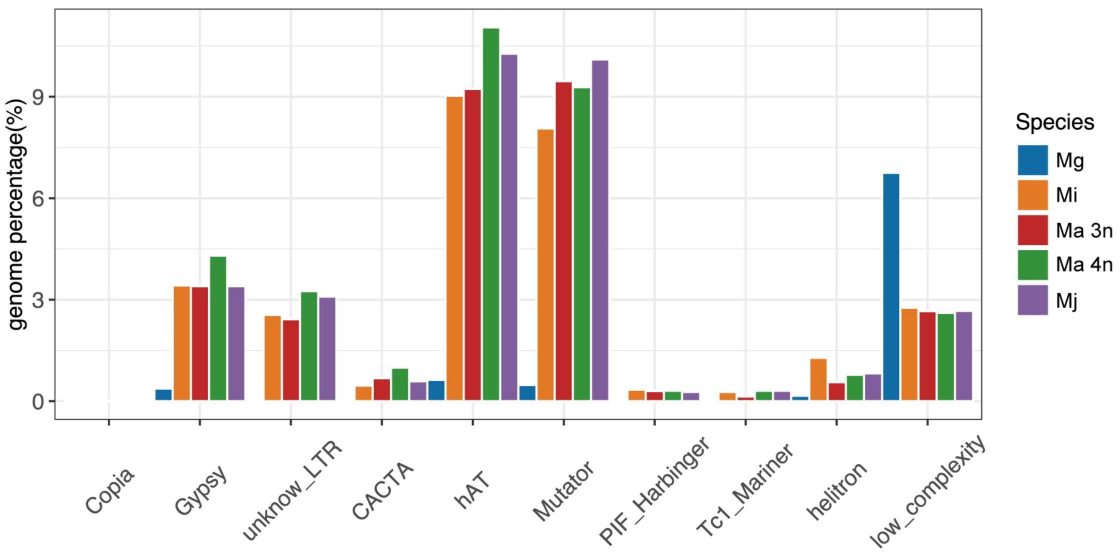

Supplementary Figure 8 Proportion of repetitive sequence in five *Meloidogyne* genome. Source data are provided as a Source Data file.

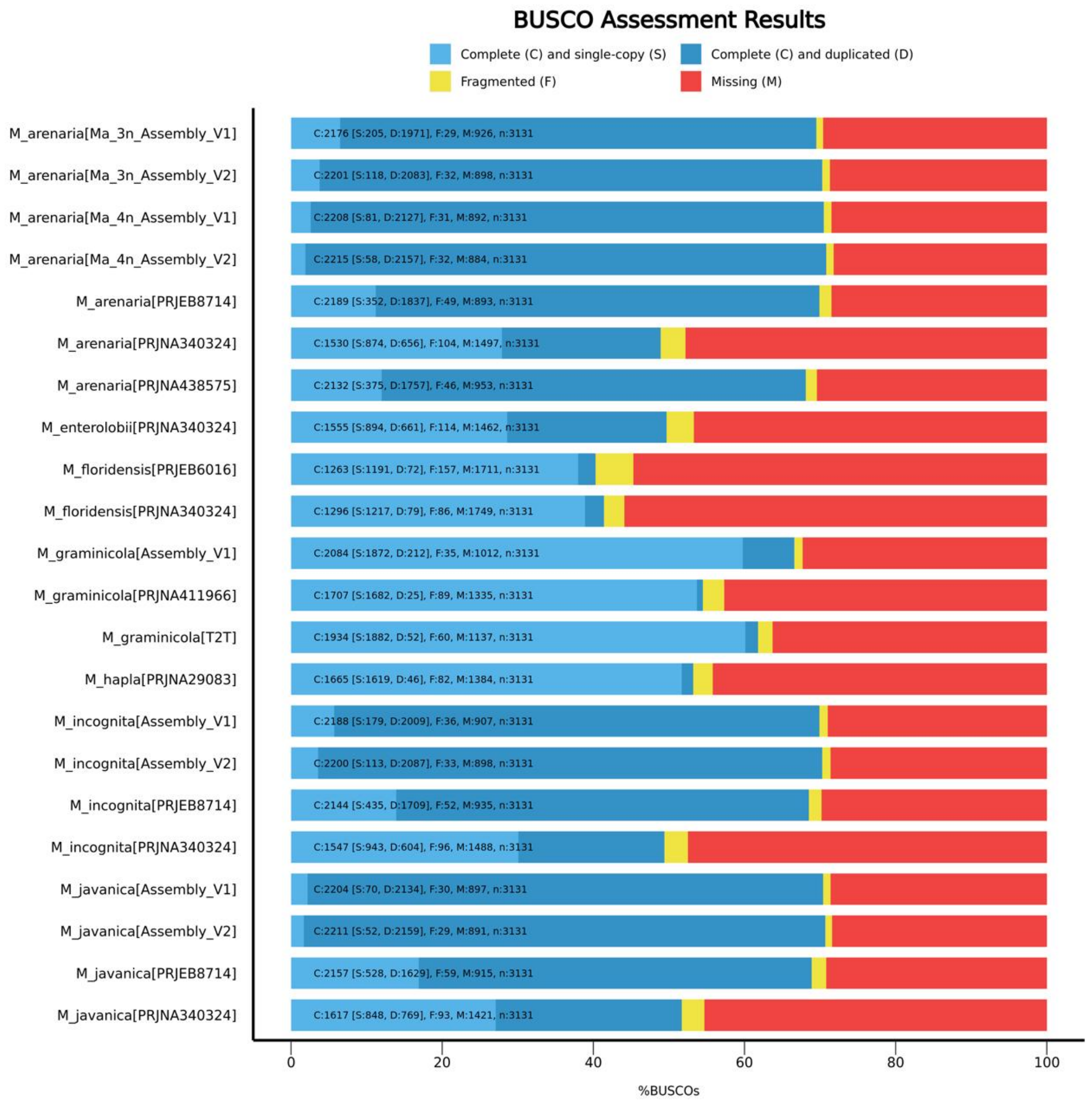

Supplementary Figure 9 BUSCO assessment for proteome. Genomes include assembly in this study and other *Meloidogyne* species genome available in Wormbase. Source data are provided as a Source Data file.

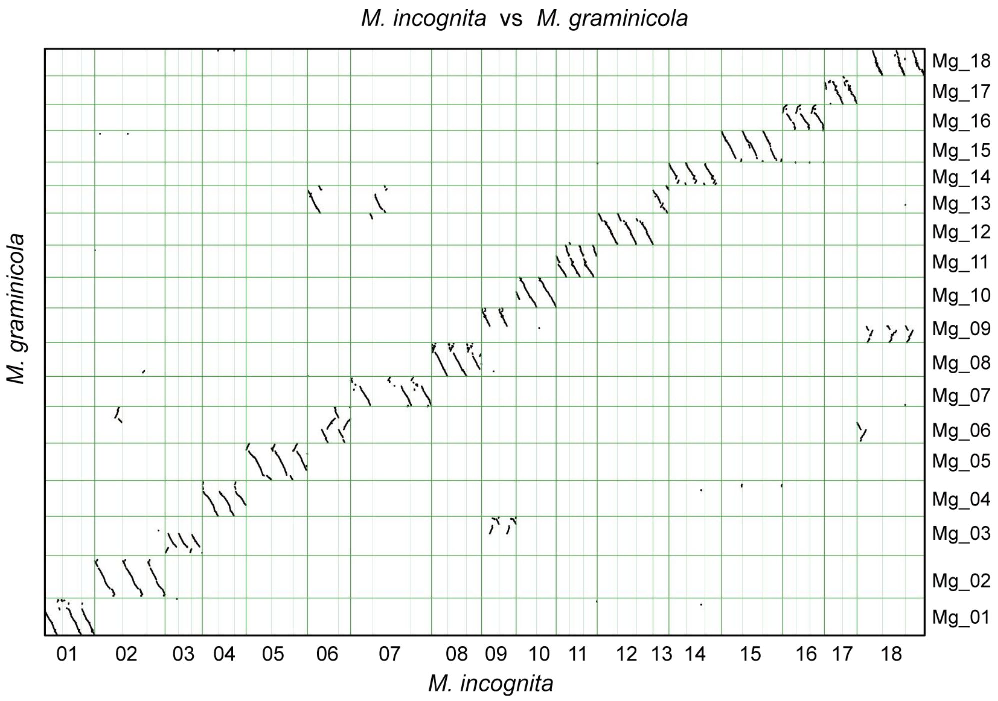

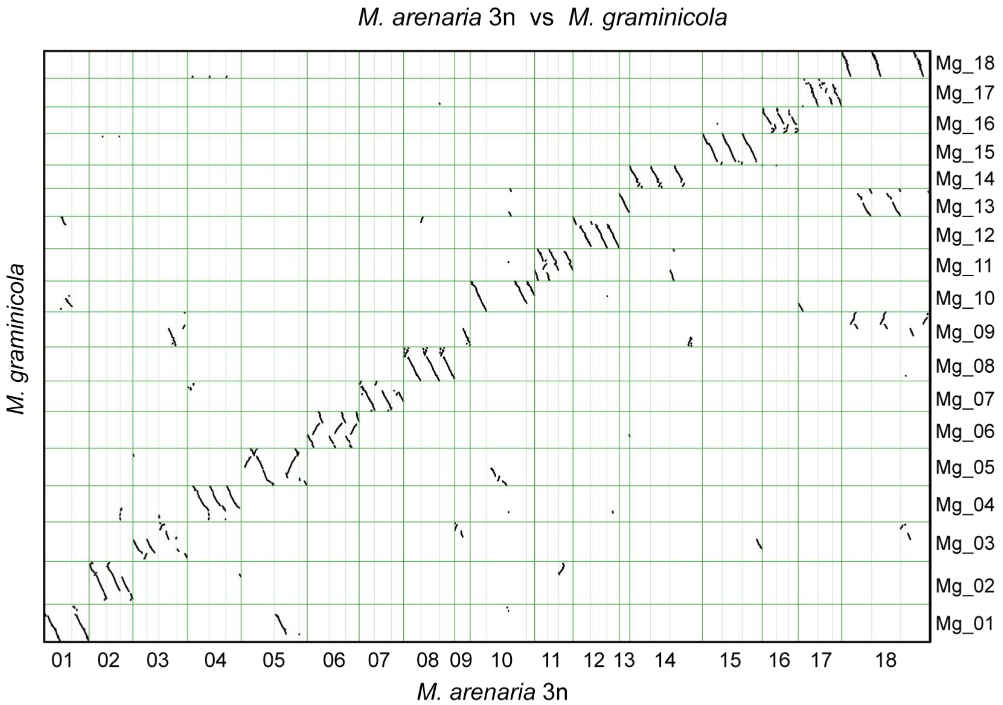

Supplementary Figure 10 Dot plot of the genomic synteny between *M. incognita* or *M. arenaria* 3n and *M. graminicola*. Syntenic relationship show a 3:1 ratio, revealing the ploidy level of *M. incognita* and *M. arenaria* 3n are triploid.

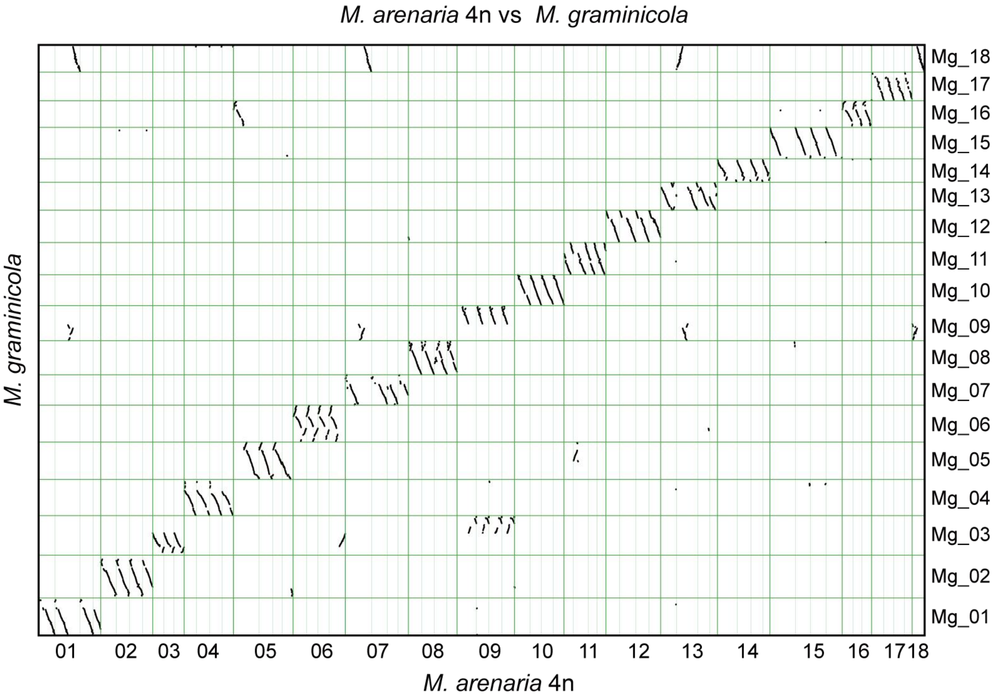

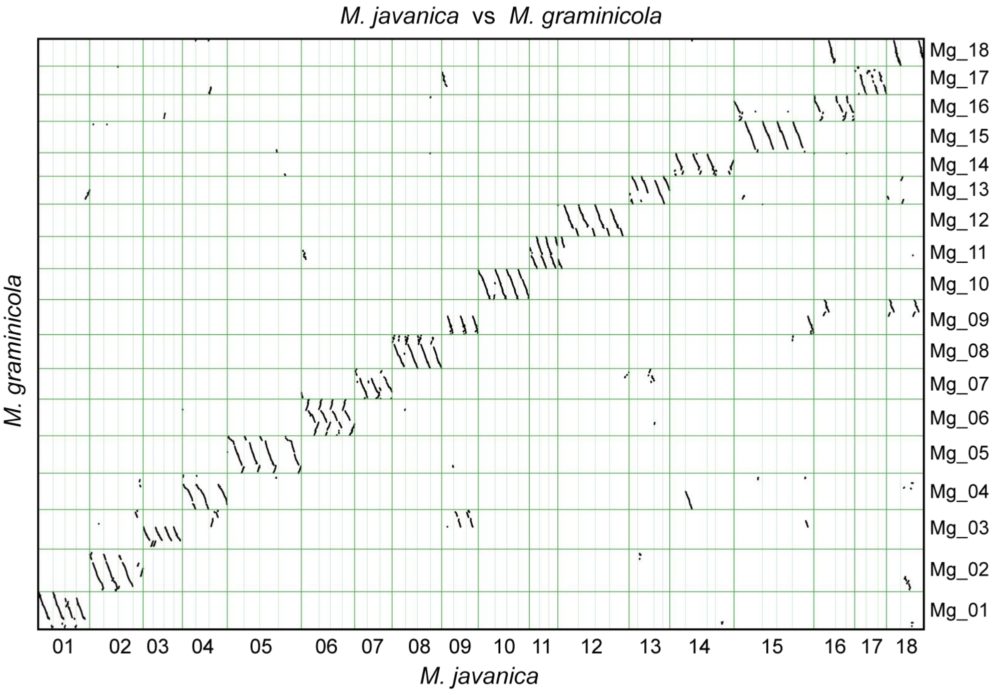

Supplementary Figure 11 Dot plot of the genomic synteny between *M. arenaria* 4n or *M. javanica* and *M. graminicola*. Syntenic relationship show a 4:1 ratio, revealing the ploidy level of *M. arenaria* 4n and *M. javanica* are tetraploid.

a

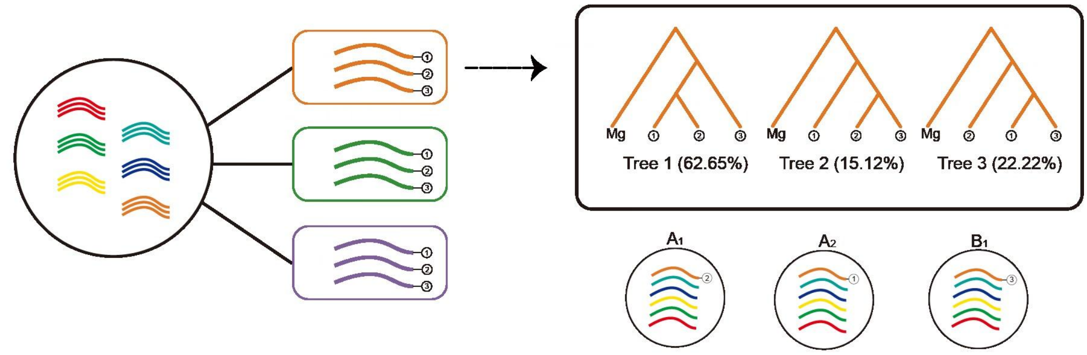

b

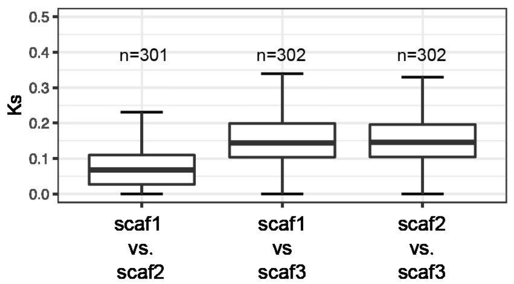

c

Nucleotide identity

	scaf_03	scaf_01	scaf_02
scaf_03	1	0.905	0.901
scaf_01	0.905	1	0.948
scaf_02	0.901	0.948	1

Supplementary Figure 12 Exploration of genome structure of triploid RKNs. (**a**) Schematic workflow of distinguishing subgenome of triploid species. Different colours represent different ancestral chromosomes. (**b**) Boxplot shows the *Ks* value between the three scaffolds of Chr01. This suggests that three homologous chromosomes of Mi are composed of two similar copies and one divergent copy. In box plots, the central line represents the median, the box represents the 25% and 75% percentiles, and the whiskers represent 1.5 times the interquartile range beyond the box. (**c**) Nucleotide identity between three scaffolds of Chr01. About 5% nucleotide divergence between two similar copies, about 10% nucleotide divergence with another divergent copy. Two similar copies are defined as A_1 and A_2, and the other is defined as B_1.

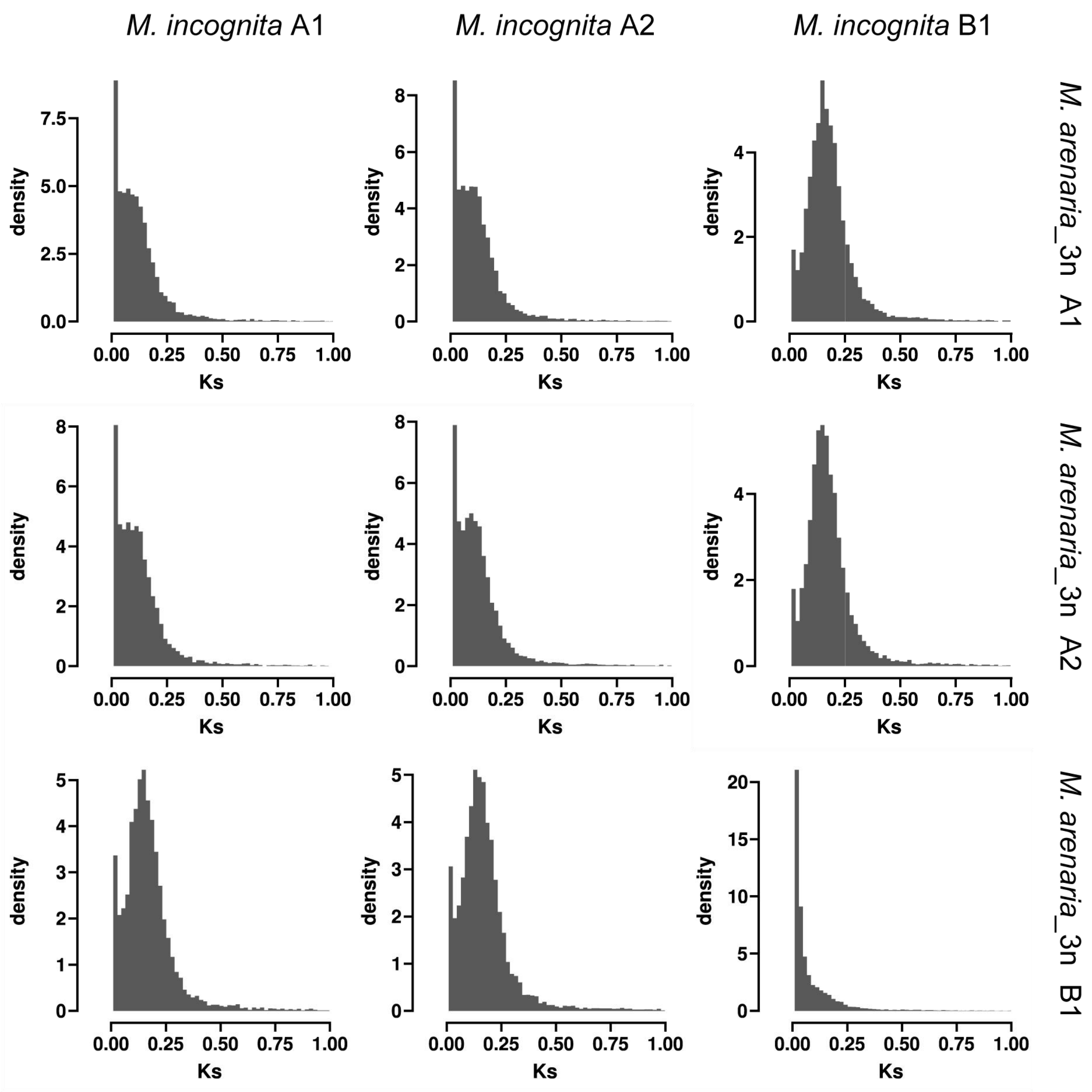

Supplementary Figure 13 The relationship between identified subgenomes of two triploids. Both triploid Mi and Ma 3n exhibit 2:1 genetic relationship (AAB or ABB). *Ks* distribution shows that both subgenome with one leaf (B) are more similar, and the subgenome with two leaves (AA) are also similar, revealing that both two triploids are AAB, instead of one AAB and one ABB. Source data are provided as a Source Data file.

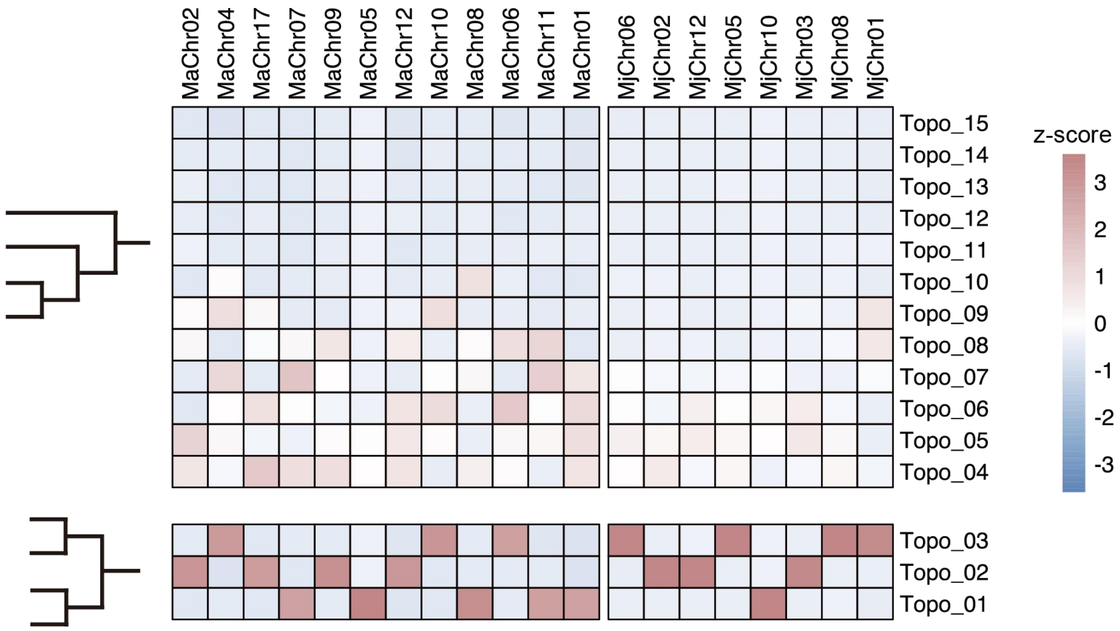

Supplementary Figure 14 Exploration of genome structure of tetraploid *Meloidogyne* species. Phylogenetic topologies of 4 homologous chromosomes for the tetraploid genome. Heatmap shows the relative number for each of 15 possible phylogenetic topologies, among which the tree with 2:2 is dominant. Source data are provided as a Source Data file.

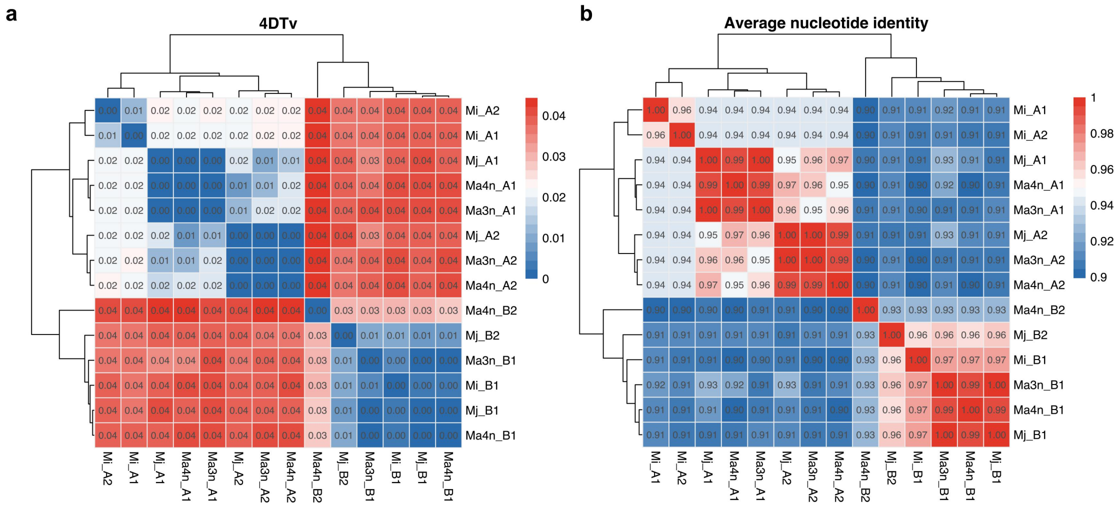

Supplementary Figure 15 Relationship between 14 subgenomes (assembly version1). (**a**) A heatmap and clustering of synonymous third codon transversion rate (4DTv). The colour intensity represents the median of 4DTv value of all syntenic gene pairs between two subgenomes. (**b**) A heatmap and clustering of nucleotide identity. The average nucleotide identity between two pair subgenomes is estimated from their scaffolds sets. This clustering is consistent with that in *Ks*. Source data are provided as a Source Data file.

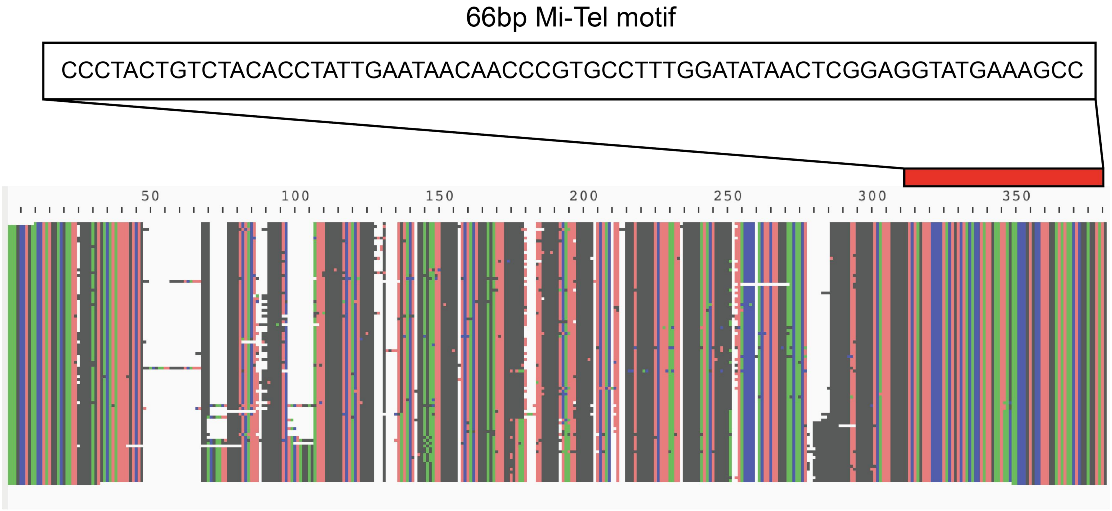

Supplementary Figure 16 Multiple sequence alignment of telomeric repeat sequences. The multiple sequence alignment is visualized using AliView (https://github.com/AliView). A 66bp conserved sequence is selected as a motif for searching Mi-Tel.

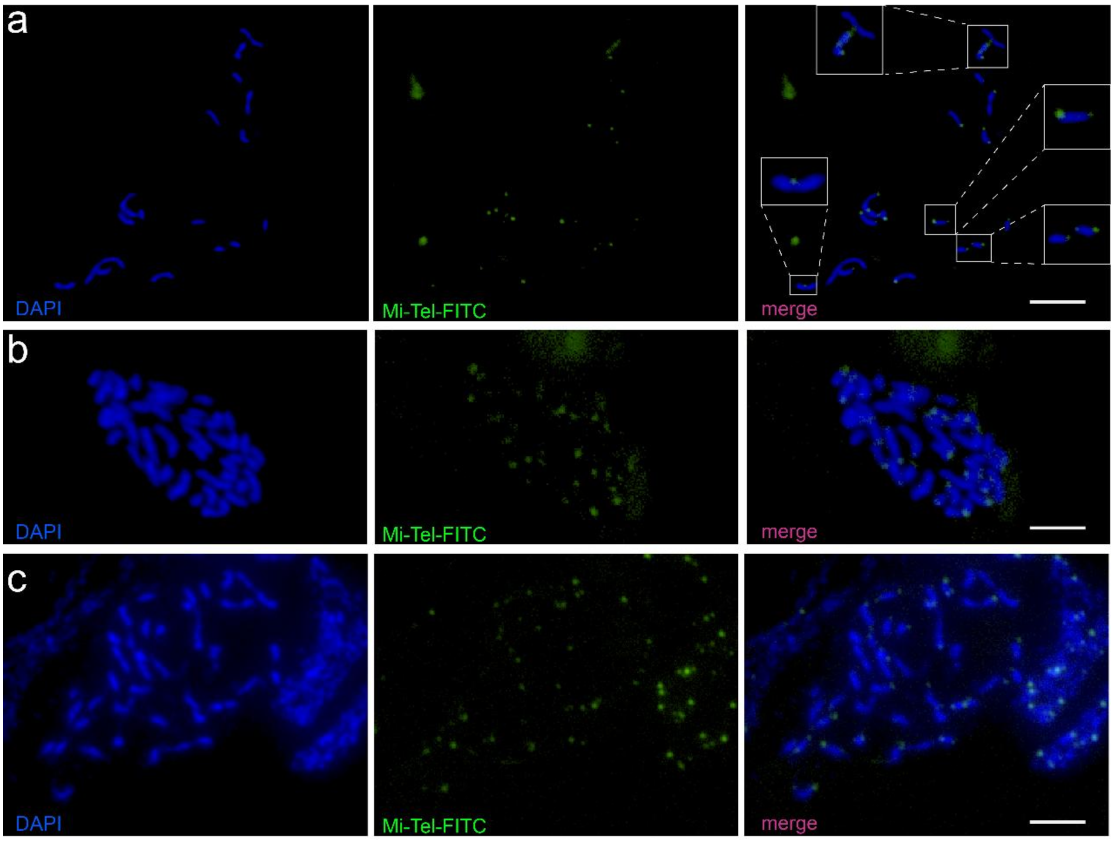

Supplementary Figure 17 Fluorescent *in situ* hybridization of Mi-Tel repeats indicated it has a terminal position on *M. incognita* chromosome. (**a**) The Mi-Tel FISH results of some Mi chromosomes are displayed. Fluorescent signals at the terminal position of multiple chromosomes can be clearly observed. Besides, a small amount of Mi-Tel single was observed to be located inside the chromosome, suggesting a possibility of telomere-to-telomere fusion between chromosomes. (**b** and **c**) The Mi-Tel FISH results of all the Mi chromosomes are displayed. Mi-Tel repeats with FITC fluorophore were used as probes for FISH, chromosomes are counterstained with DAPI, scale bar = 5 μm. Four independent FISH experiments on Mi-Tel all yielded similar results.

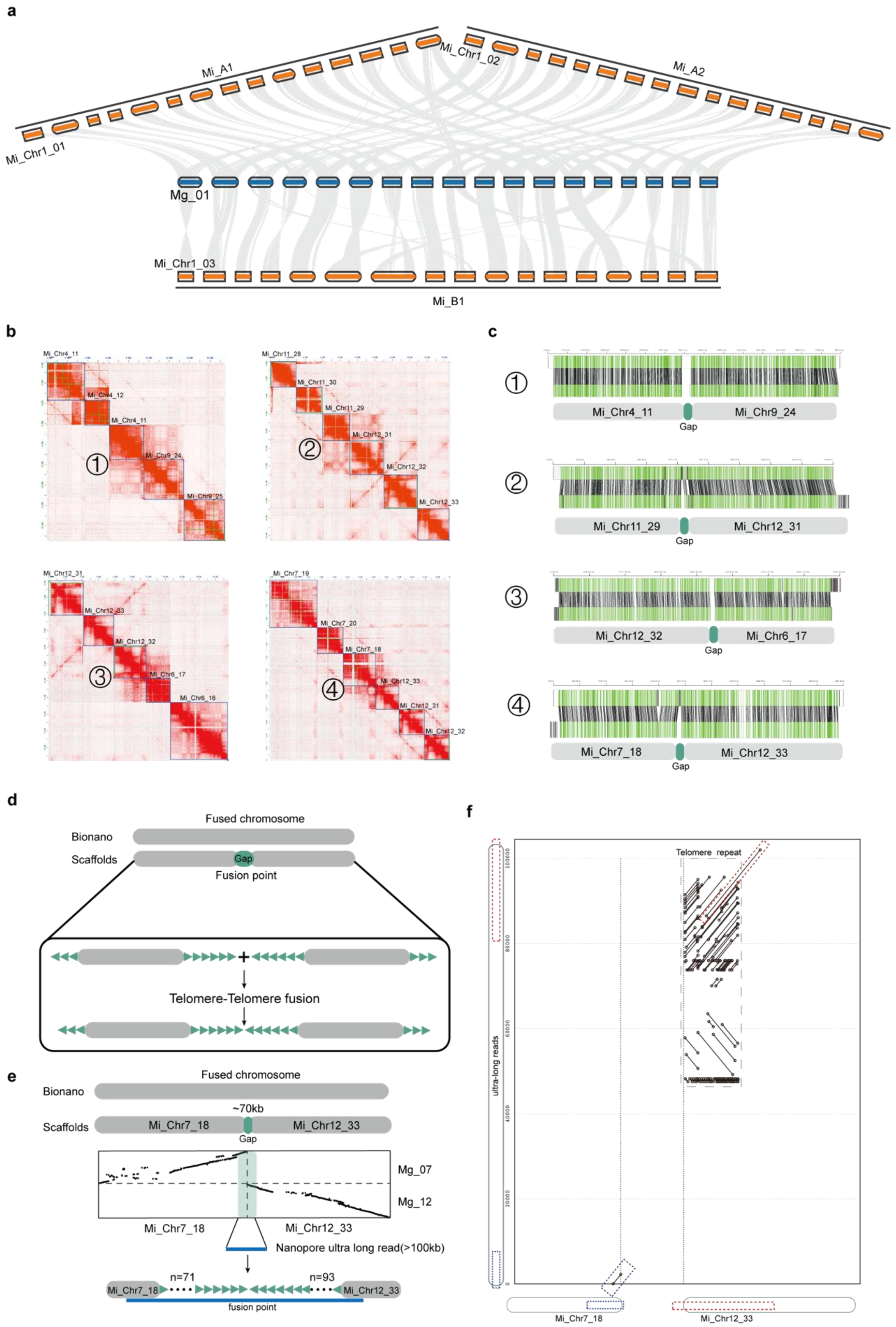

Supplementary Figure 18 Putative telomere-to-telomere fusion events in *M. incognita*. (**a**) The nomenclature for scaffolds of assembly v1. For example, three scaffolds that exhibit collinearity with Mg_01 are named as Mi_Chr1_01, Mi_Chr1_02, and Mi_Chr1_03. (**b**)-(**c**) Evidence for putative chromosomal fusion.

The assembly v1 refer to their collinearity with Mg genome to some extent, thus many fused chromosomes are split into 2-3 scaffolds. Heatmap on the left shows the originally Hi-C signal between those scaffolds. In BioNano alignment on the right, the upper track is BioNano consensus map, the bottom track is scaffolds in assembly v1. We determined chromosomal fusion events as that there is an obvious Hi-C signal between the two scaffolds and a BioNano consensus map is completely aligned with these two scaffolds. (**d**) Schematic diagram of the telomere-to-telomere fusion hypothesis. (**e**) Evidence for chromosome telomere-to-telomere fusion. BioNano map between two Mi scaffolds (Mi_Chr7_18 and Mi_Chr12_33) shows a 70 kb gap. The blue line indicates the nanopore ultralong reads spanning two chromosomes. (**f**) Dot plot between ultra-long read and two fused scaffolds. Only the 50kb region at the end of the scaffolds are shown. This ONT long read (@4a68ac45-6400-4c5d-8b2d-02d12dd19299) spans the fusion point of two ancestral chromosomes. The black and red indicates the matches between scaffolds sequences and this long read.

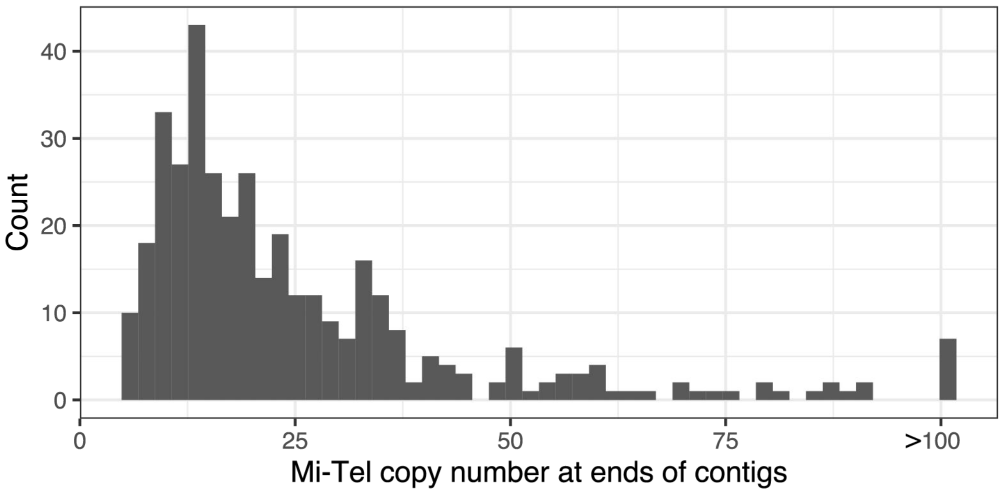

Supplementary Figure 19 Copy number of Mi-Tel at telomere. At the ends of contigs, the average copy number of Mi-Tel is 26, with a median of 19. Source data are provided as a Source Data file.

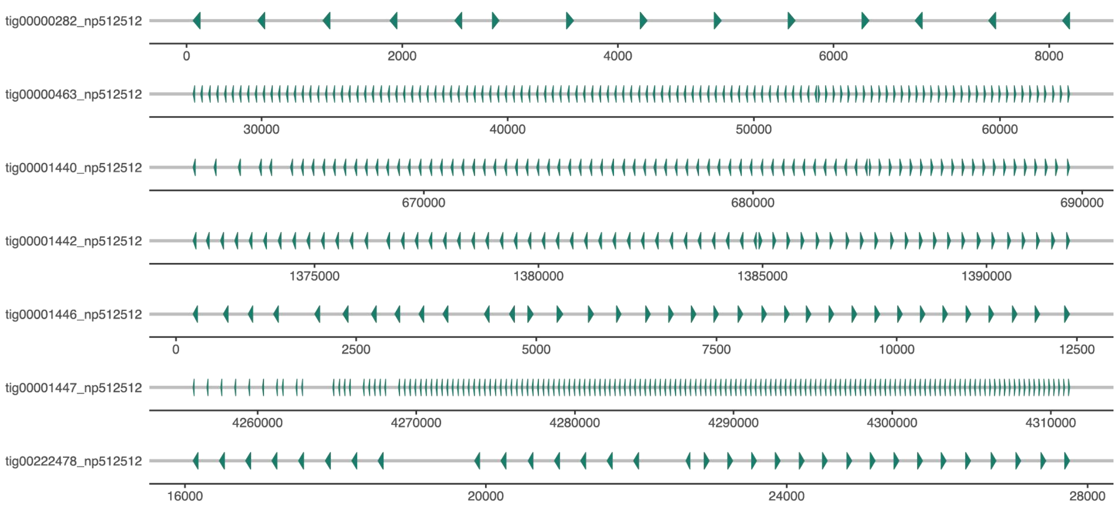

Supplementary Figure 20 Short contigs that contain symmetrically distributed Mi-Tel.

a

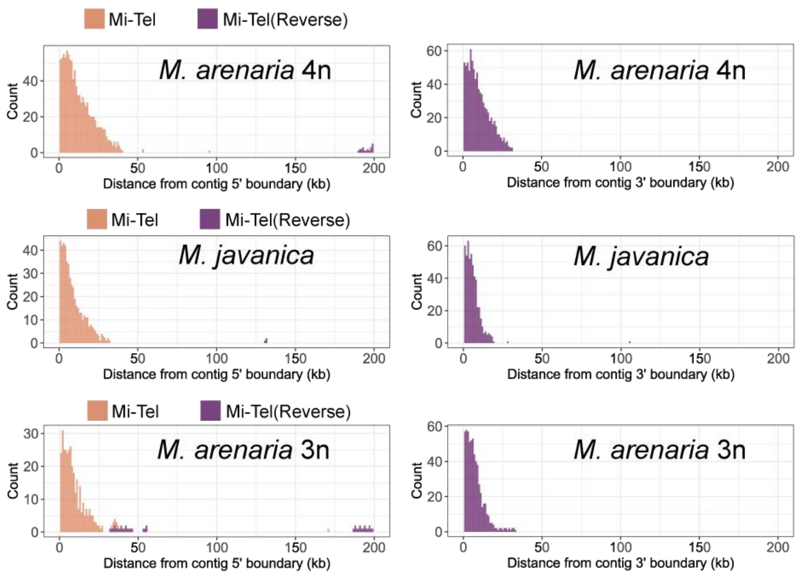

b

Species	Mi-Tel	
Meloidogyne chitwoodi	0	-
Meloidogyne graminicola	0	-
Meloidogyne exigua	0	-
Meloidogyne hapla	0	-
Meloidogyne floridensis_1	21	+
Meloidogyne floridensis_2	29	+
Meloidogyne enterolobii	1315	+
Meloidogyne luci	1107	+

Supplementary Figure 21 Conservation of telomeric repeats in other *Meloidogyne*. (**a**) Distribution of telomeric repeats in Ma 4n, Mj and Ma 3n in this study. Only contigs larger than 200kb are shown. (**b**) Number of telomeric repeats (Mi-Tel) in other *Meloidogyne* species. The *M. chitwoodi* (GCA_015183035.1), *M. exigua* (GCA_018905775.1), *M. hapla* (GCA_000172435.1), *M. floridensis_1* (GCA_003693605.1), *M. floridensis_2* (GCA_000751915.1), *M. enterolobii* (GCA_903994135.1) and *M. luci* (GCA_902706615.1) genome are download from NCBI. Source data are provided as a Source Data file.

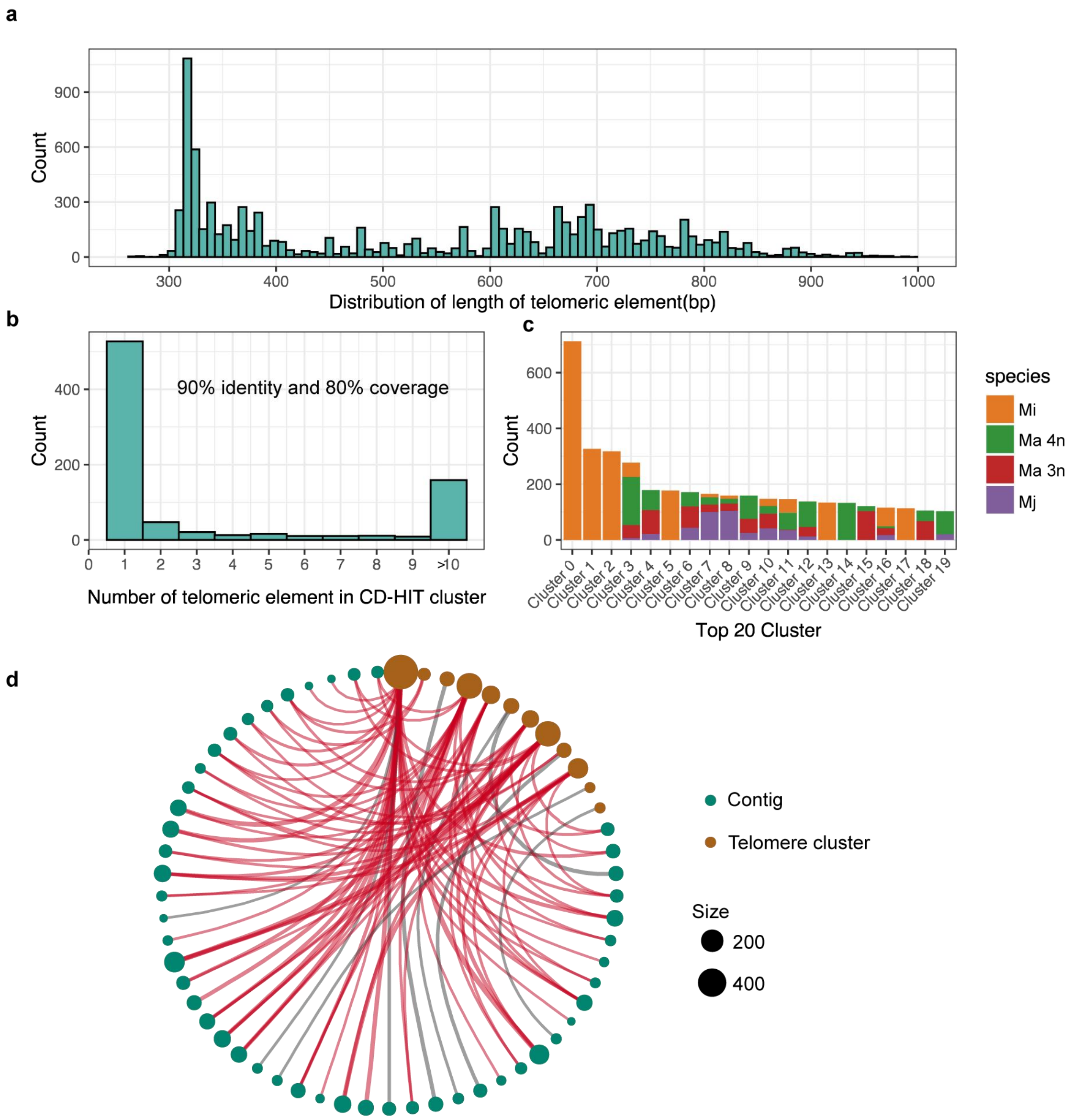

Supplementary Figure 22 Features of telomeric element for polyploid *Meloidogyne* species. (**a**) Distribution of length of 9318 telomeric elements. (**b**) Distribution of cluster size of each CD-HIT cluster. Sequences with an identity greater than 90% and a coverage greater than 80% will be clustered together. (**c**) Species composition for top 20 CD-HIT clusters. (**d**) Distribution pattern of telomere clusters in contigs. Red line indicates that there are multiple clusters of telomere element on the same contig. Source data are provided as a Source Data file.

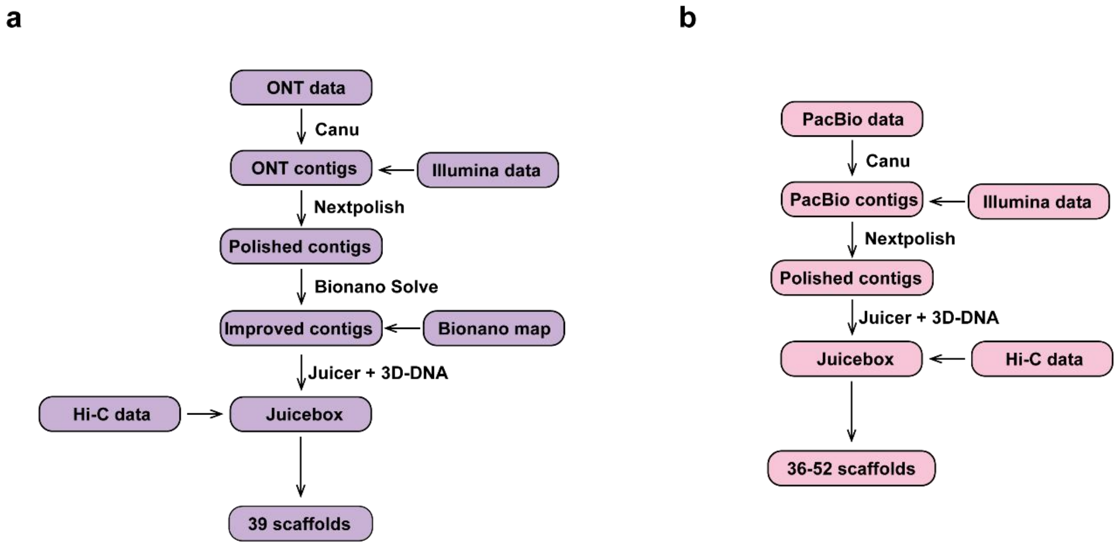

Supplementary Figure 23 Workflow of chromosome-level genome construction for Mi (a) and other 3 polyploid RKNs (b).

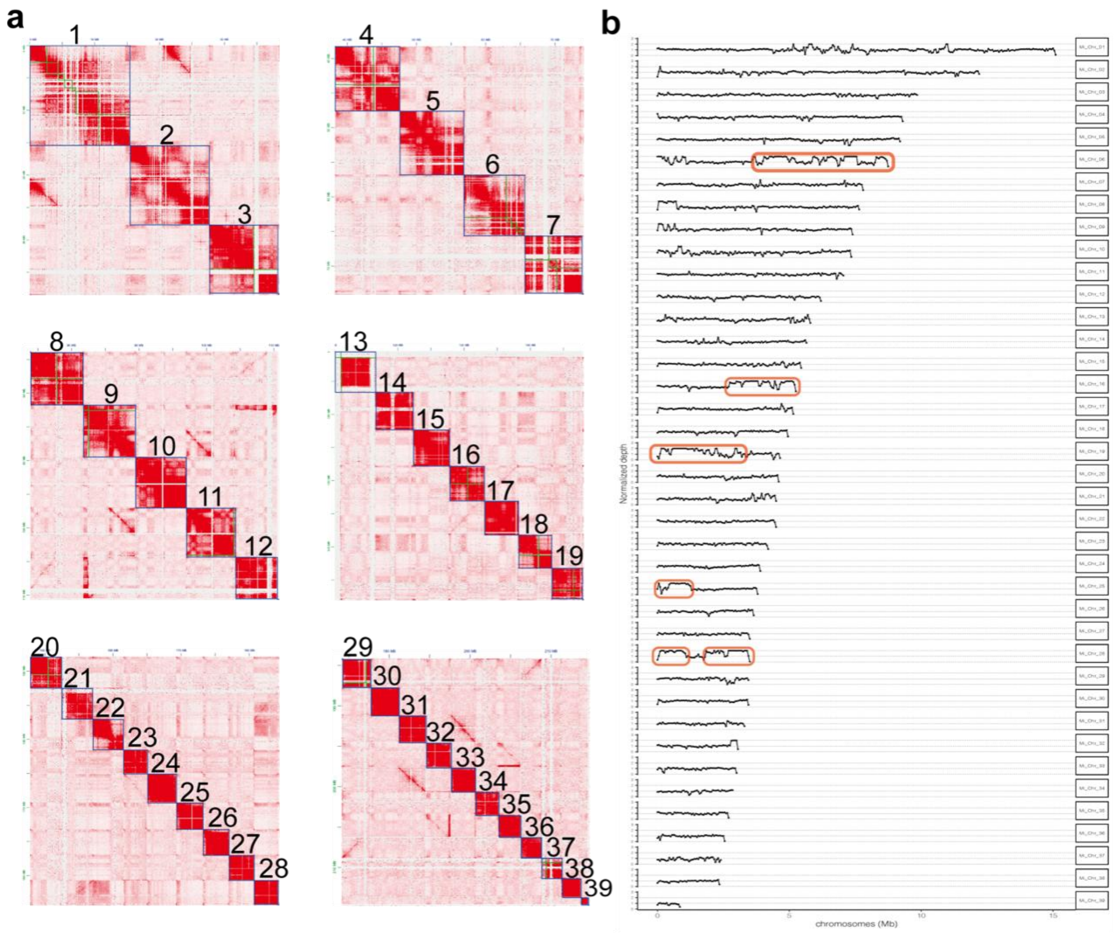

Supplementary Figure 24 Chromosome-level assembly for *M. incognita*. (**a**) Genome-wide Hi-C heatmap in Juicebox. (**b**) Distribution of read depths among chromosomes. The normalized depth is calculated in 100kb windows. The large-scale collapsed regions are marked in red. Source data are provided as a Source Data file.

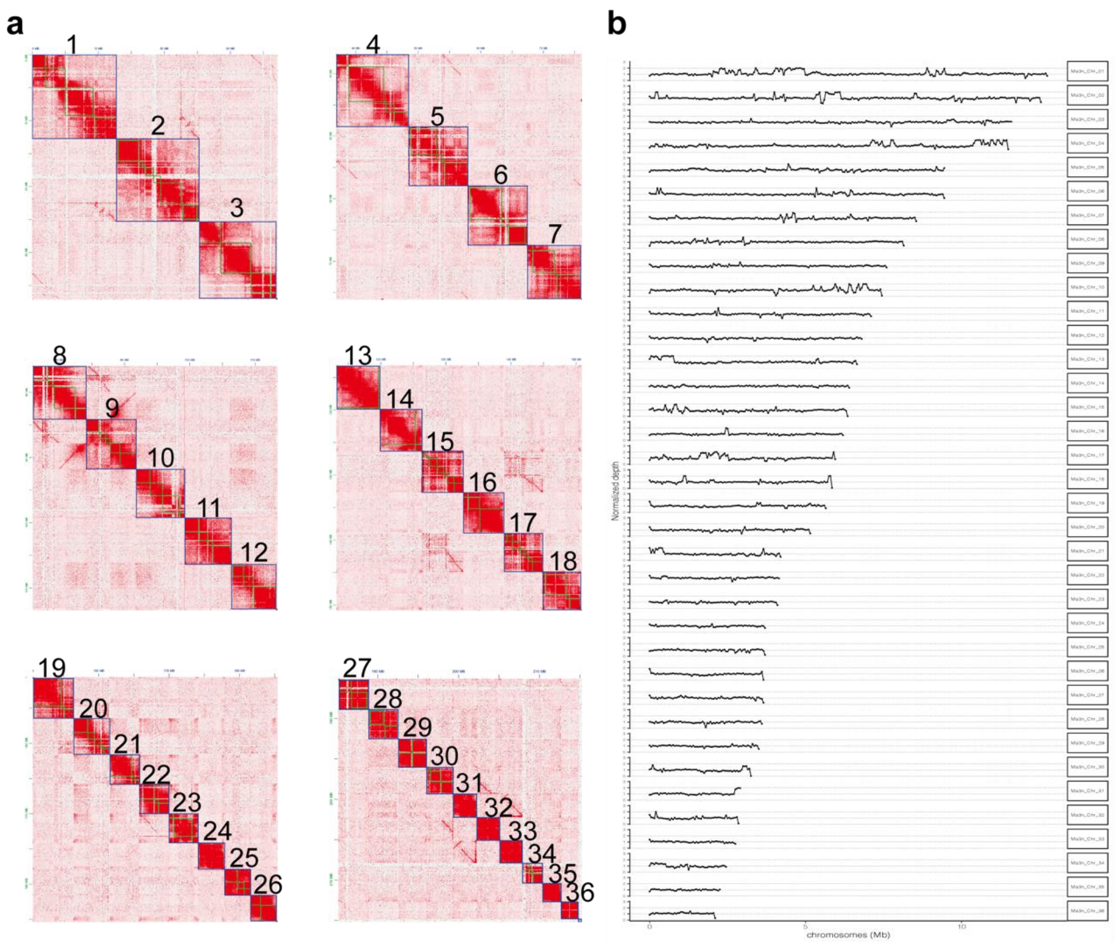

Supplementary Figure 25 Chromosome-level assembly for *M. arenaria* 3n. (**a**) Genome-wide Hi-C heatmap in Juicebox. (**b**) Distribution of read depths among chromosomes. The normalized depth is calculated in 100kb windows. Source data are provided as a Source Data file.

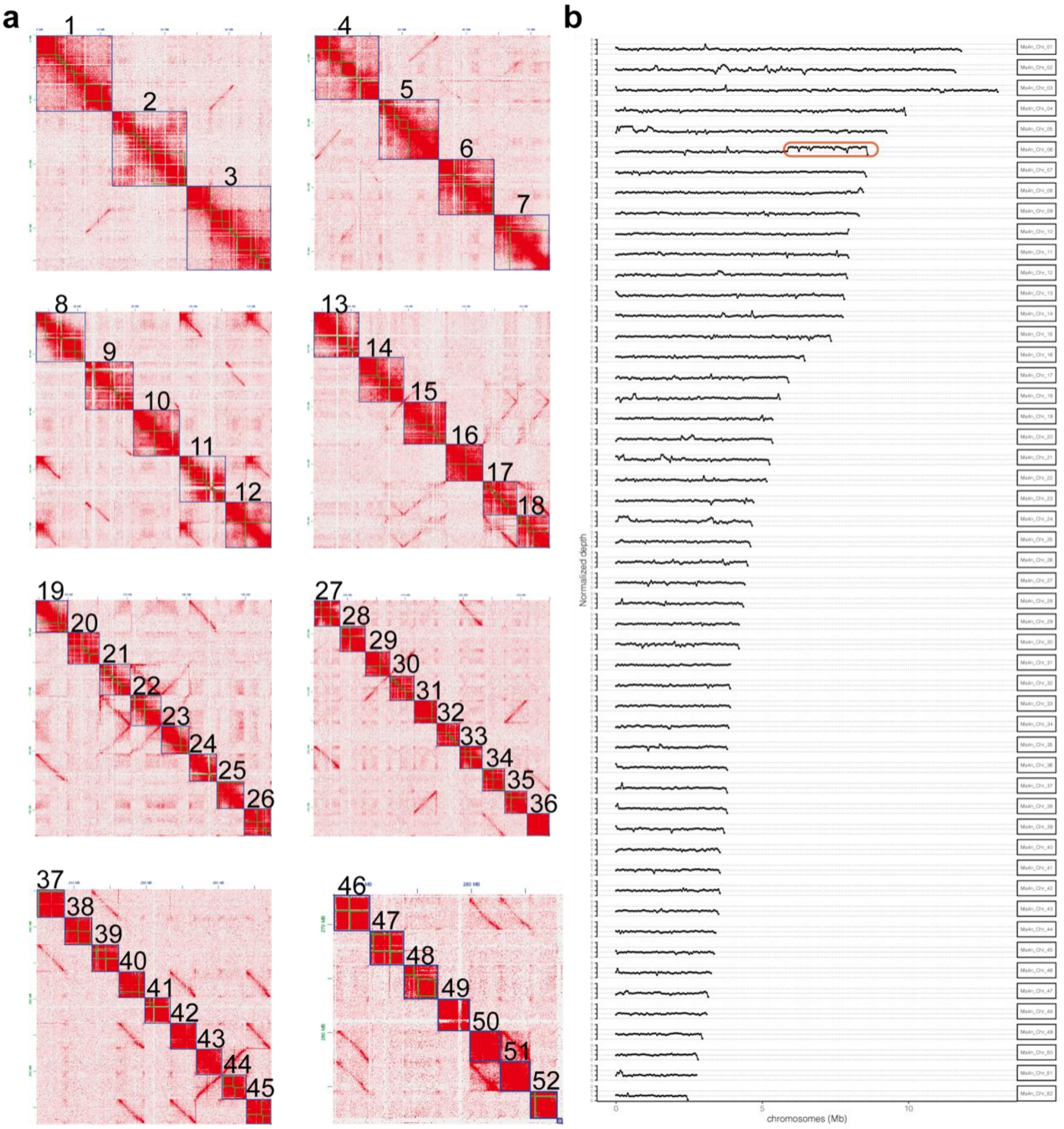

Supplementary Figure 26 Chromosome-level assembly for *M. arenaria* 4n. (**a**) Genome-wide Hi-C heatmap in Juicebox. (**b**) Distribution of read depths among chromosomes. The normalized depth is calculated in 100kb windows. The large-scale collapsed region is marked in red. Source data are provided as a Source Data file.

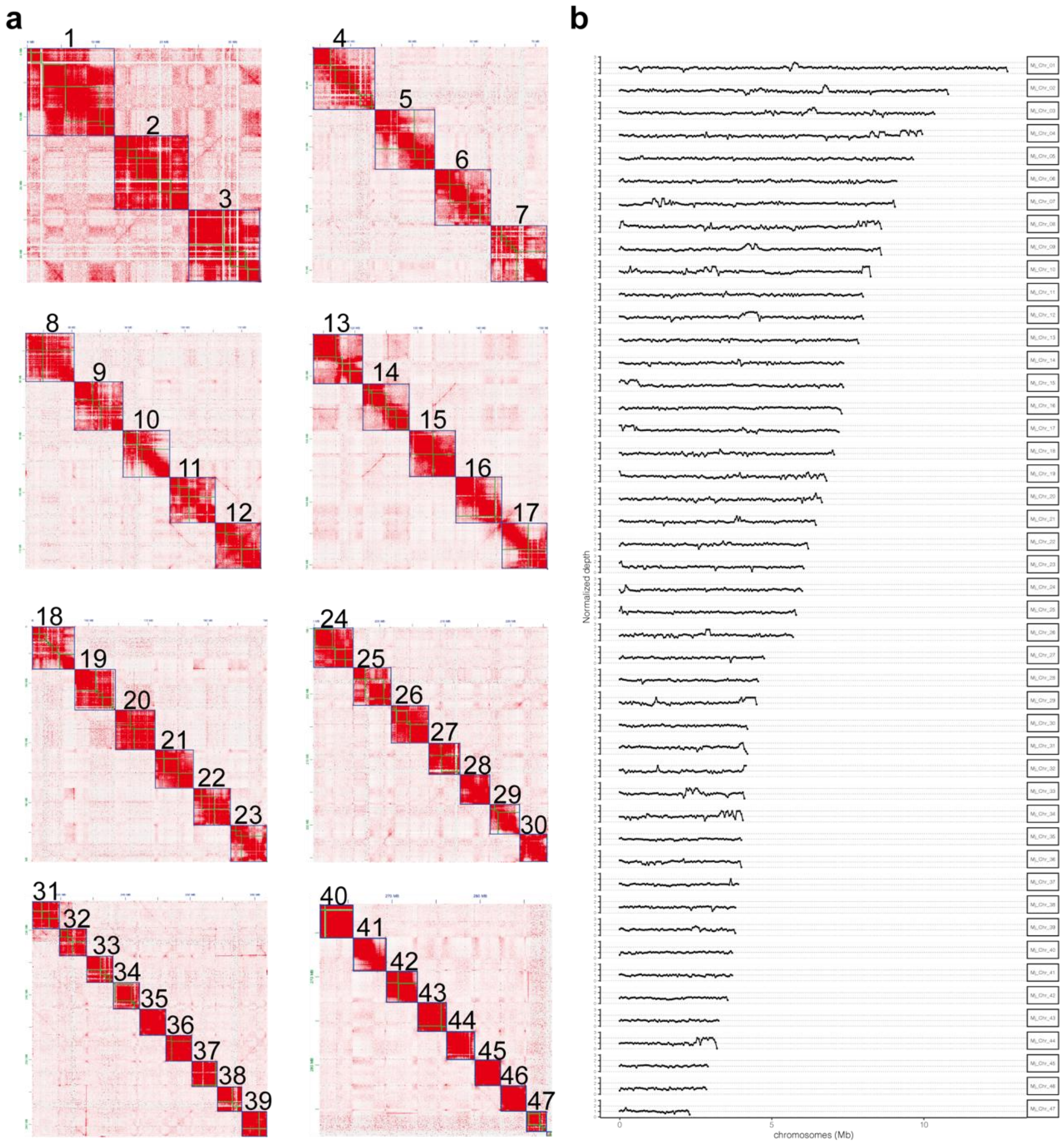

Supplementary Figure 27 Chromosome-level assembly for *M. javanica*. (**a**) Genome-wide Hi-C heatmap in Juicebox. (**b**) Distribution of read depths among chromosomes. The normalized depth is calculated in 100kb windows. The large-scale collapsed region is marked in red. Source data are provided as a Source Data file.

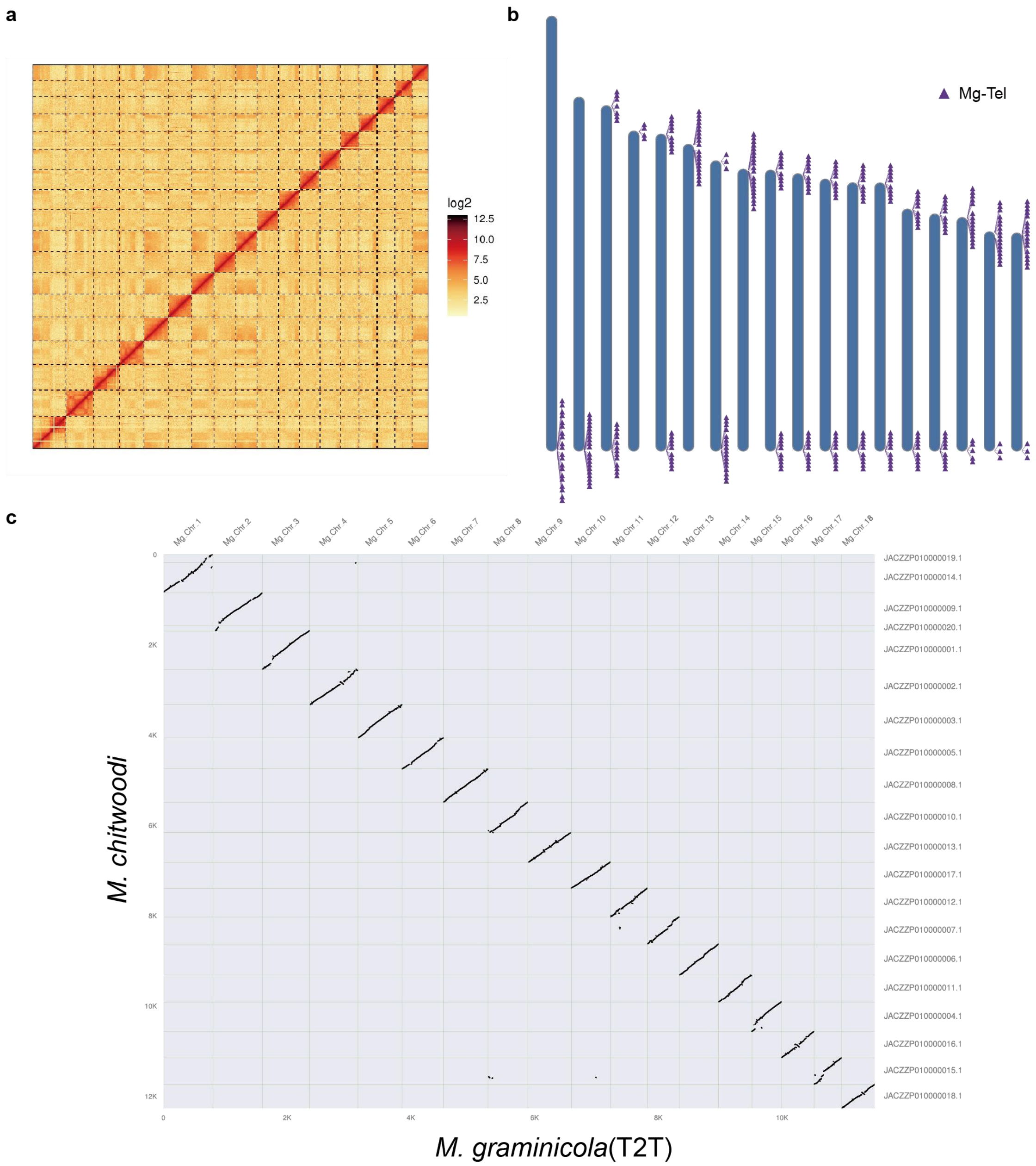

Supplementary Figure 28 Construction of T2T genome for *M. graminicola.* (**a**) Genome-wide Hi-C heatmap of Mg T2T genome. (**b**) Distribution of Mg-Tel among 18 chromosomes of Mg T2T genome. (**c**) Genomic synteny of Mg T2T with *M. chitwoodi* genome (GCA_015183035.1). Only scaffolds larger than 500kb are shown.

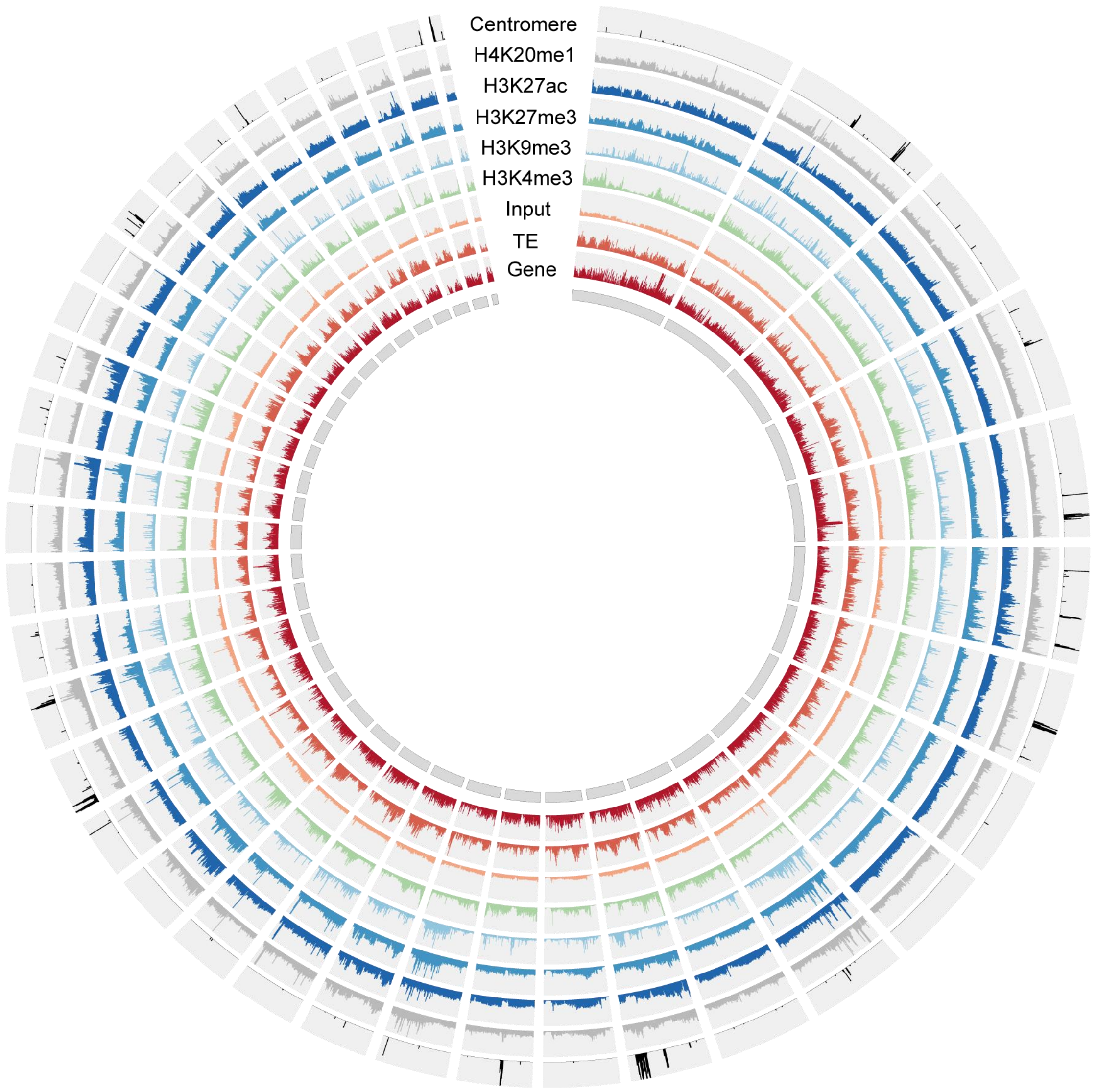

Supplementary Figure 29 Distribution of genetic elements across *M. incognita* chromosomes. Circos plot shows genomic features of *M. incognita*. The tracks show the gene density, TE density, ChIP-seq input signal, H3K4me3 density, H3K9me3 density, H3K27me3 density, H3K27ac density, H4K20me1 density, and centromere motif density. All of features are counted in 50kb windows. Source data are provided as a Source Data file.

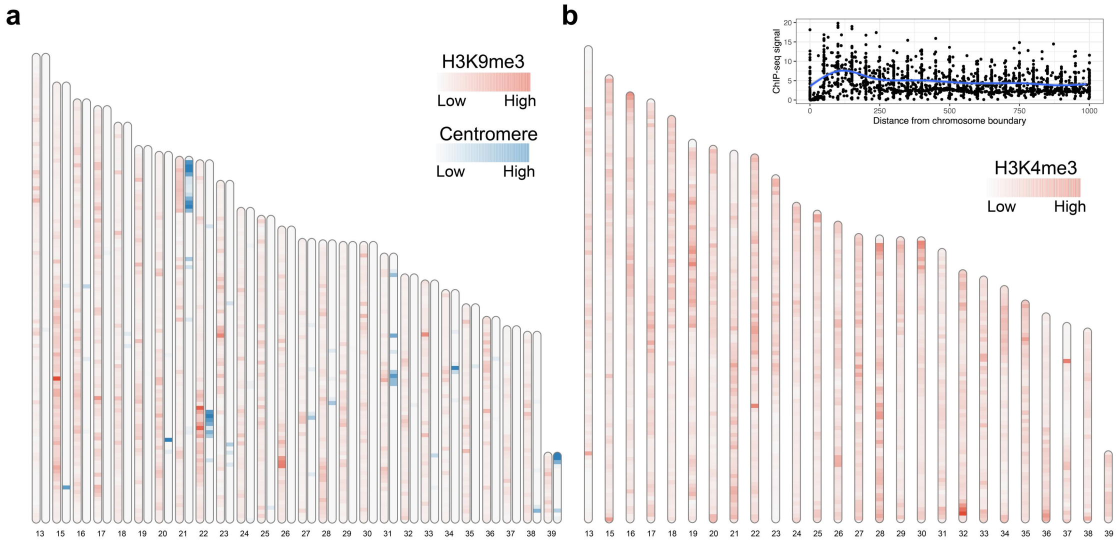

Supplementary Figure 30 The relationship between histone modification and telomere and centromere. (**a**) Distribution of H3K9me3 in relation to centromere. (ChIP-seq signal for centromeric region: 7.6; whole genome: 2.9, Mann-Whitney $P < 0.01$) (**b**) Distribution of H3K4me3 in relation to sub-telomeric region. (ChIP-seq signal for sub-telomeric region: 4.8; whole genome: 3.9, Mann-Whitney $P < 0.01$) Only chromosomes without fusion are shown. Source data are provided as a Source Data file.

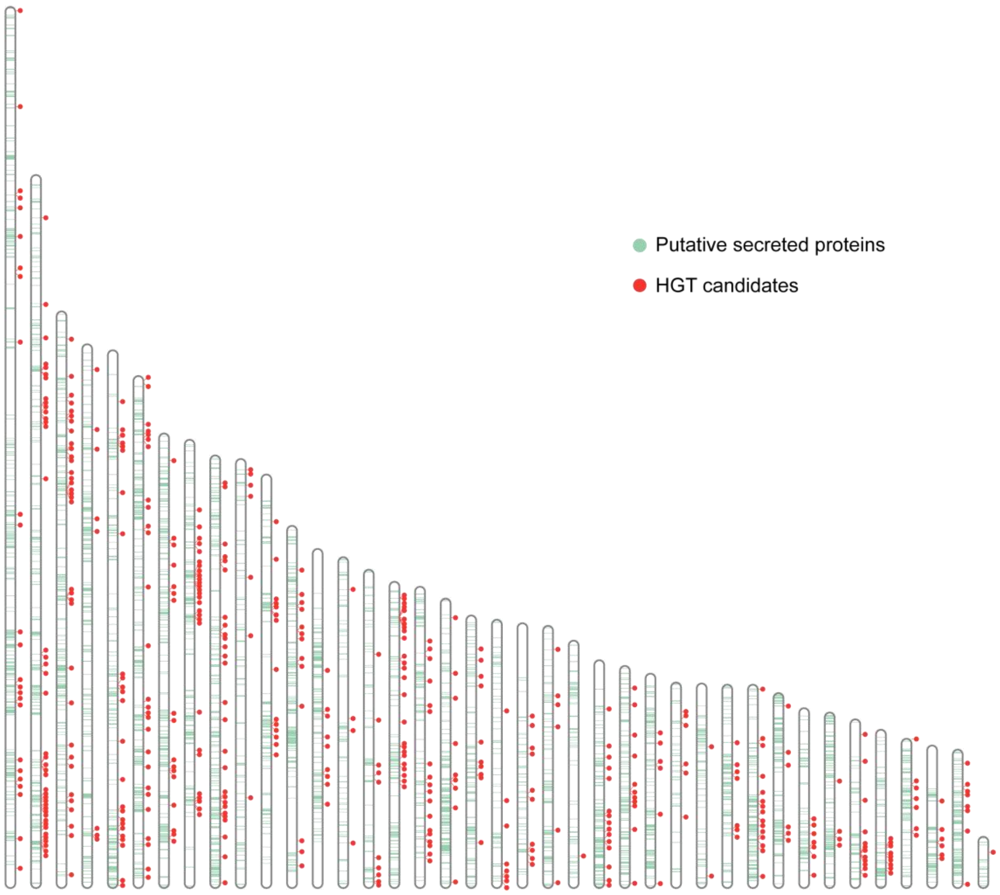

Supplementary Figure 31 Distribution of HGT candidates and putative secreted protein coding genes in *M. incognita*.

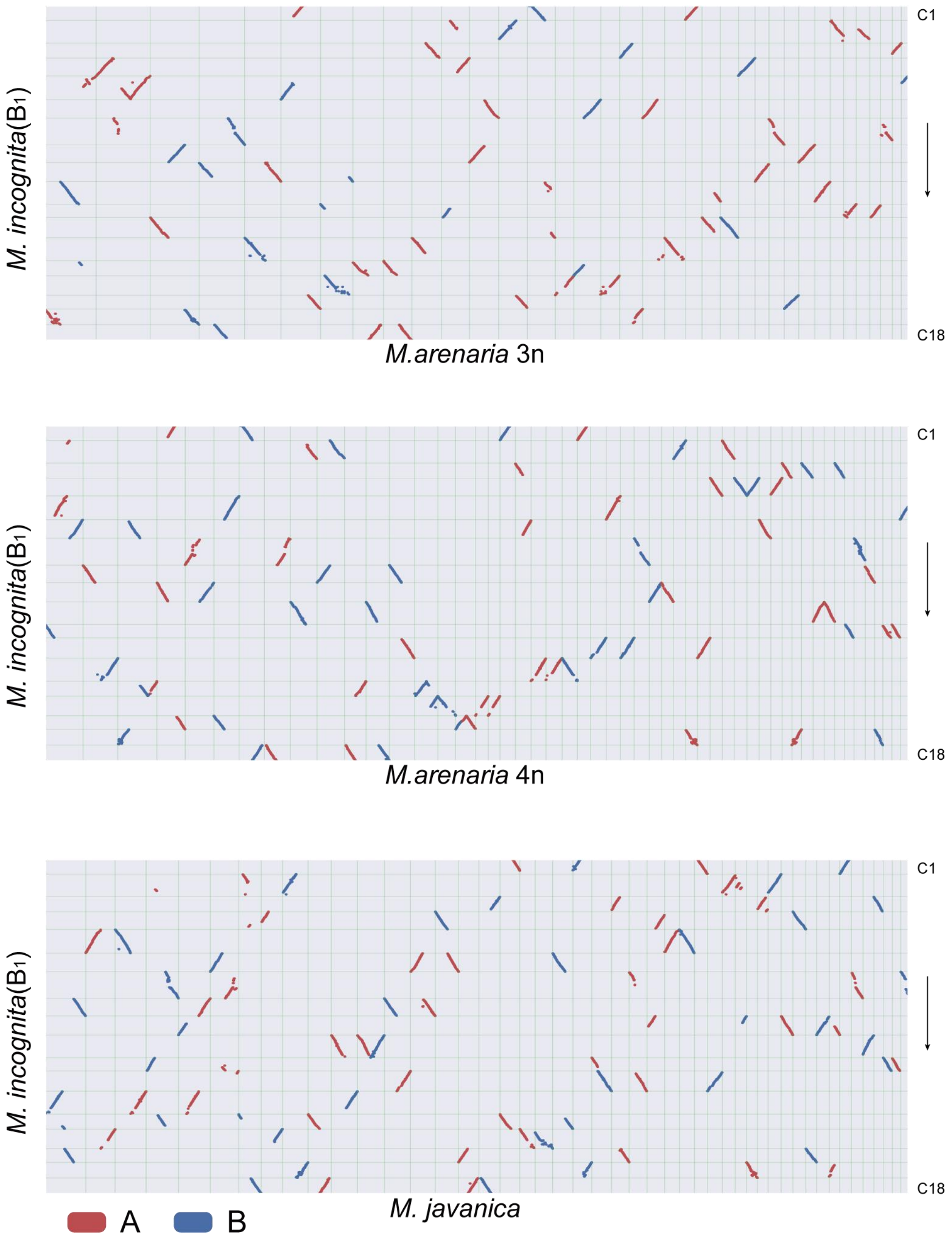

Supplementary Figure 32 Genomic synteny of *M. arenaria* 3n, *M. arenaria* 4n and *M. javanica* with *M. incognita* B_1 subgenome. The A_1 or A_2 subgenome are marked as red, and B_1 or B_2 subgenome are marked as blue. The A or B subgenome are inferred by *Ks* value between them and B_1 subgenome, where the lower one as B, the higher one as A subgenome.

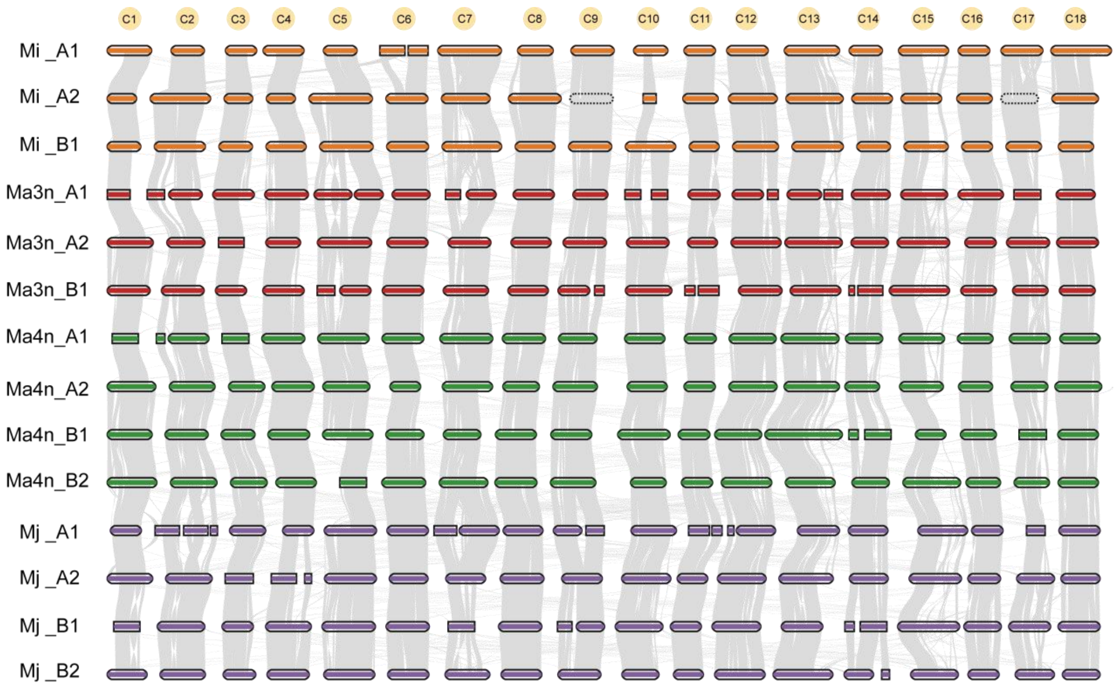

Supplementary Figure 33 Genomic synteny of 14 subgenomes belonging to polyploid species (assembly v2). Grey line indicates the collinearity between two subgenomes. The region with low synteny is likely the centromeric region. The A_1 and A_2 subgenome in here are assigned randomly. The chromosome set C1 - C18 are defined according to synteny with Mi B_1 genome

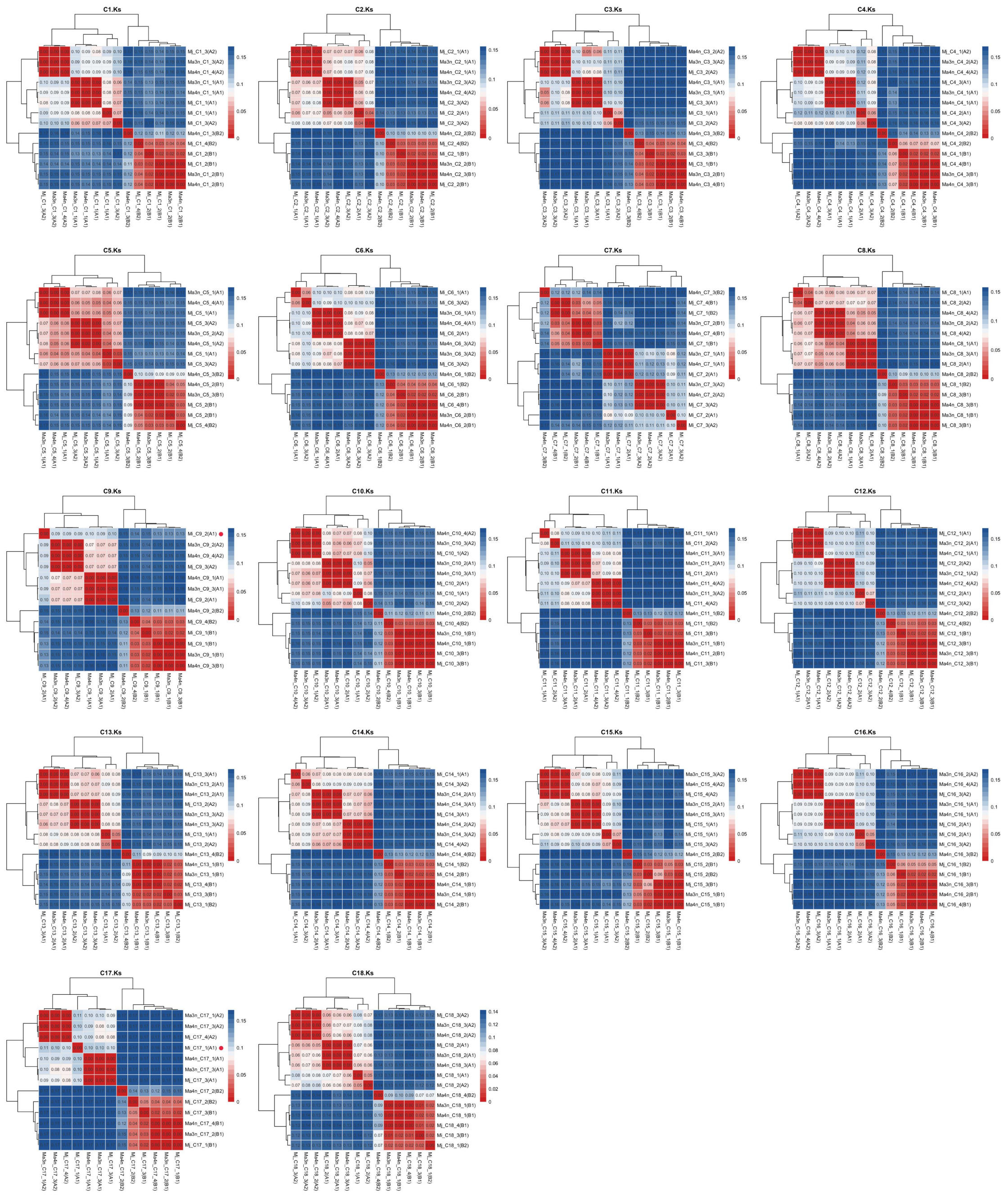

Supplementary Figure 34 Ks matrix of 14 subgenomes for 18 chromosome sets. Each heatmap and clustering represents the relationship of subgenomes belonging to an ancestral chromosome set. Based on known Mi B_1 subgenome, those heatmap are used to infer subgenomes of the other chromosome. The red dots represent the sequences that have been collapsed from A_1 and A_2. Source data are provided as a Source Data file.

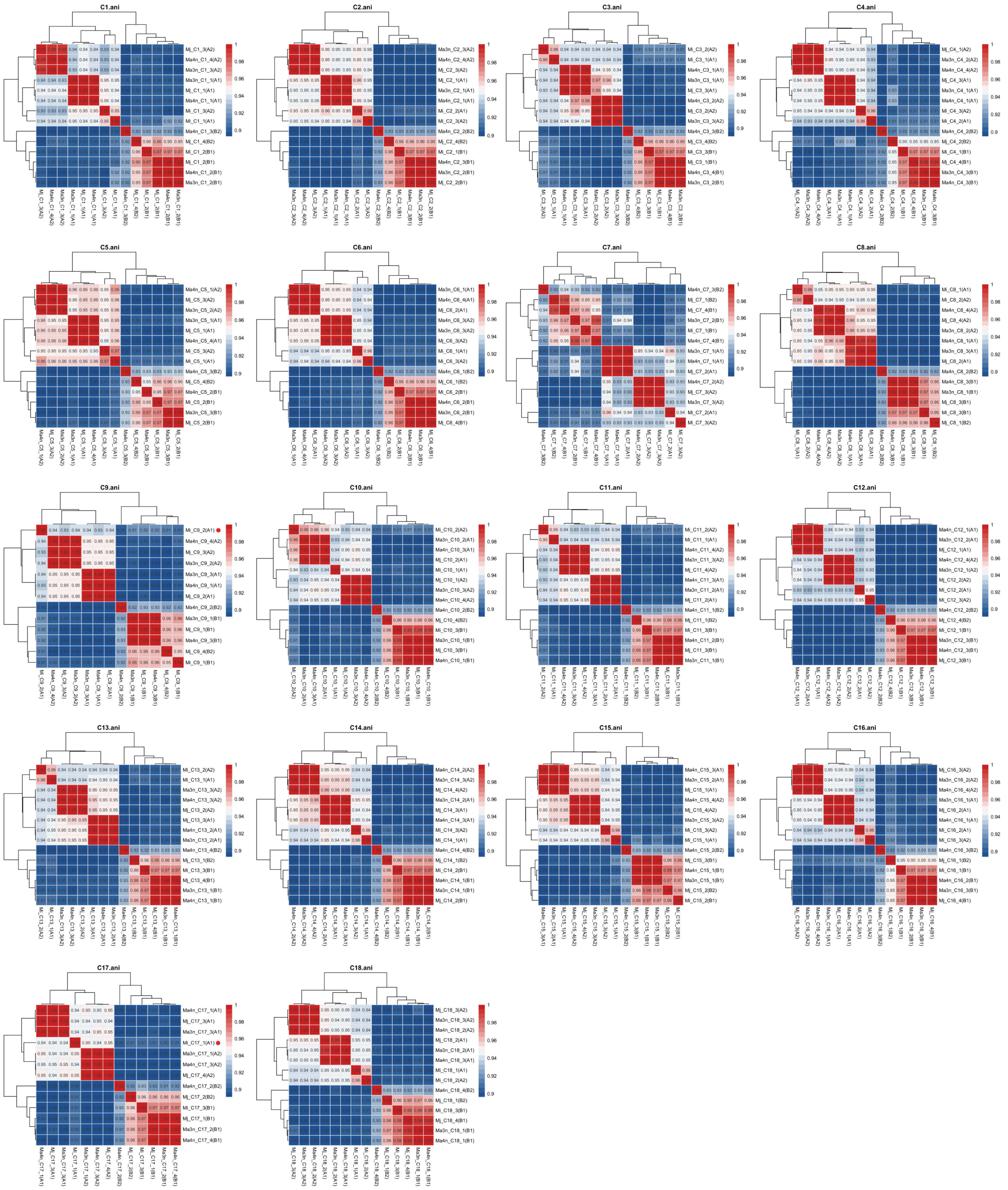

Supplementary Figure 35 Nucleotide identity of 14 subgenomes for 18 chromosome sets. Each heatmap and clustering represents the relationship of subgenomes belonging to an ancestral chromosome set. The red dots represent the sequences that have been collapsed from A_1 and A_2. Source data are provided as a Source Data file.

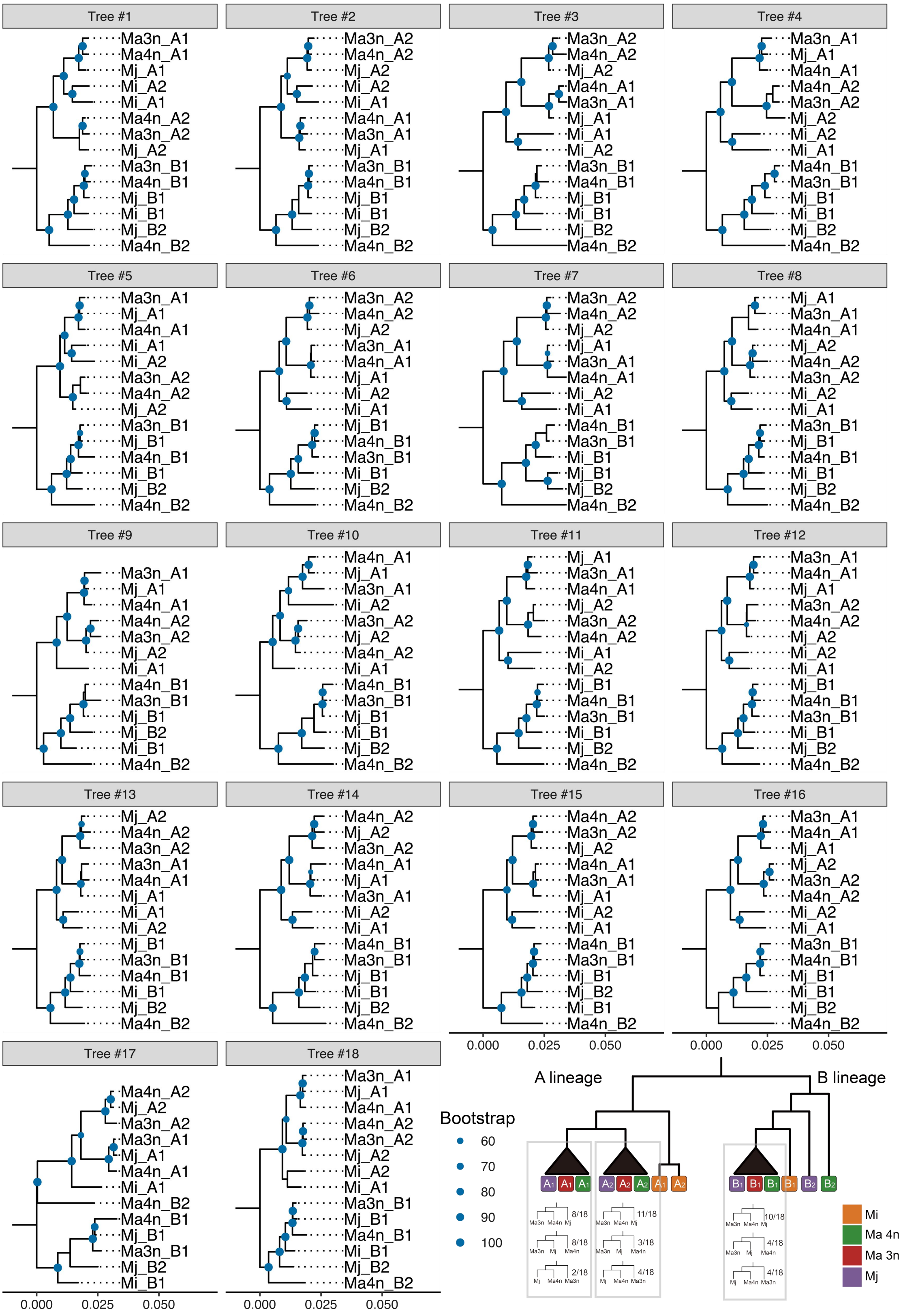

Supplementary Figure 36 Phylogenetic tree of 14 subgenomes for 18 chromosome sets. Each maximum-likelihood tree represents a phylogeny of 14 subgenomes belonging to an ancestral chromosome set. These 18 trees are used to infer reticulate the origin of polyploid species. Trees are rooted by midpoint.

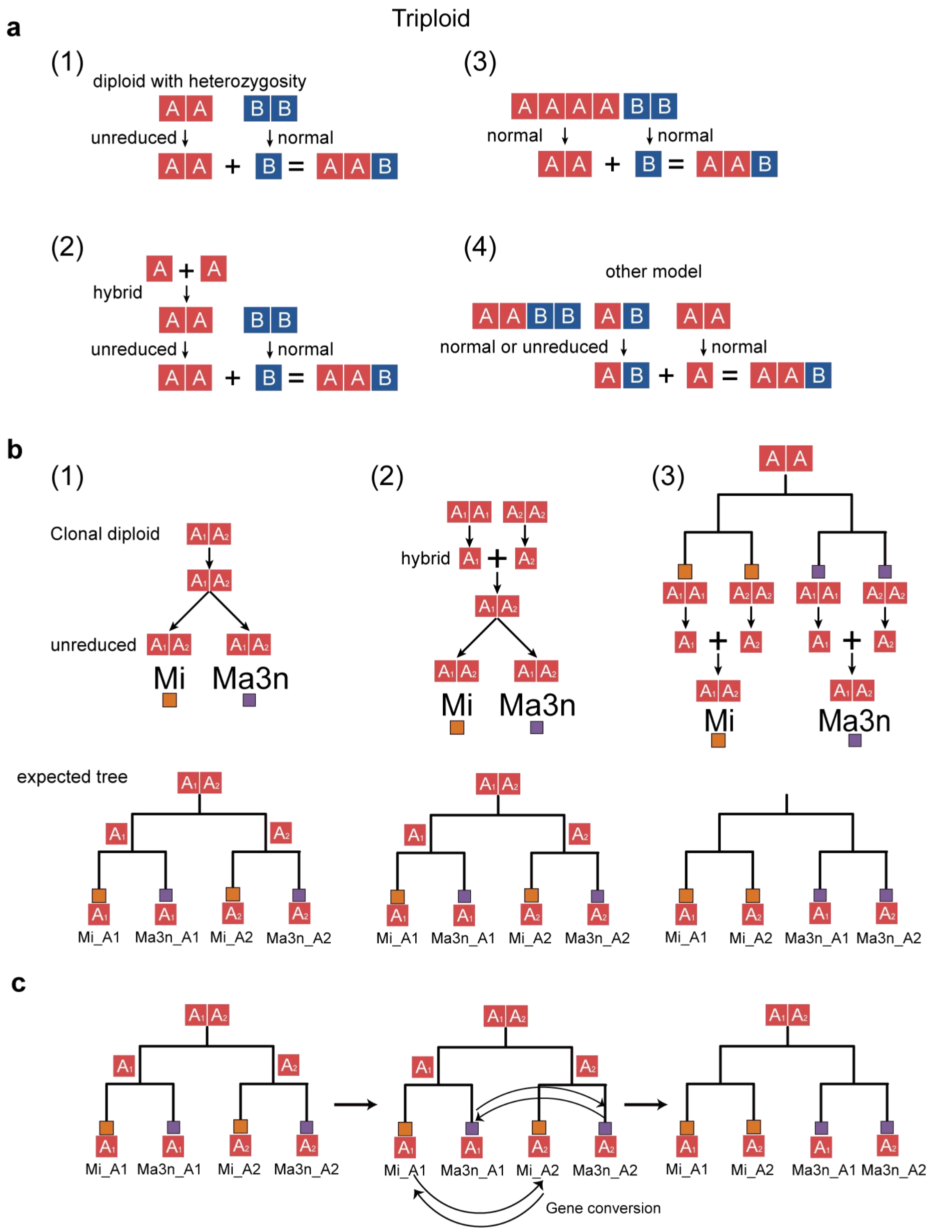

Supplementary Figure 37 Formation hypothesis of triploid species. (**a**) Scenarios for triploid species formation from A and B ancestor. (**b**) Scenarios for AA gametes formation of two triploids and the expected phylogenetic topology. (**c**) Gene conversion explains why the tree topology is inconsistent with our model.

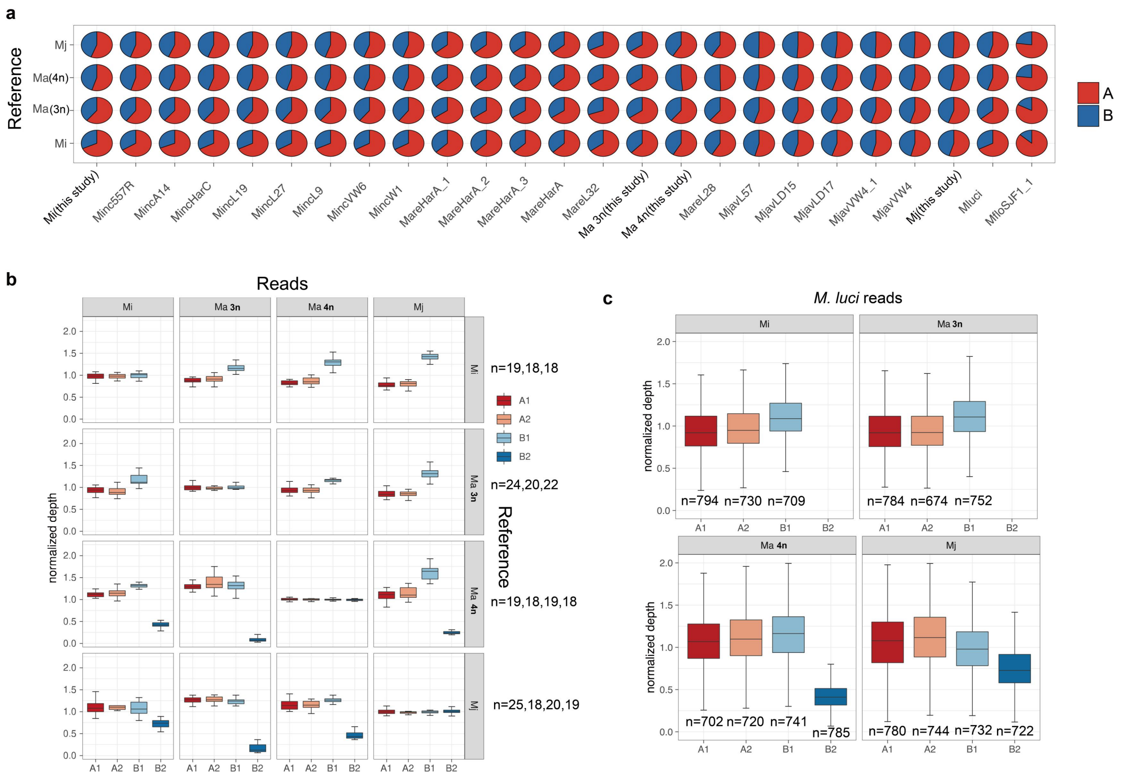

Supplementary Figure 38 Evidence for genome structure of *M. floridensis* and *M. luci.* (**a**) Proportion of reads belonging to A or B subgenome. Short reads of each *Meloidogyne.* accessions are mapped into four polyploid genome. Each pie chart represents the ratio of reads belonging to A and B subgenome of this alignment. Regardless of which reference genome used, most of *M. floridensis* reads (76%-86%) are assigned into A subgenome. (**b**) Boxplot of normalized reads depth for polyploid species in this study. (**c**) Boxplot of normalized reads depth for *M. luci*. Pattern of alignment obtained from *M. luci* data are similar with those in *M. incognita*. These data are used to infer genome structure of *M. floridensis* and *M. luci*. In box plots, the central line represents the median, the box represents the 25% and 75% percentiles, and the whiskers represent 1.5 times the interquartile range beyond the box. Source data are provided as a Source Data file.

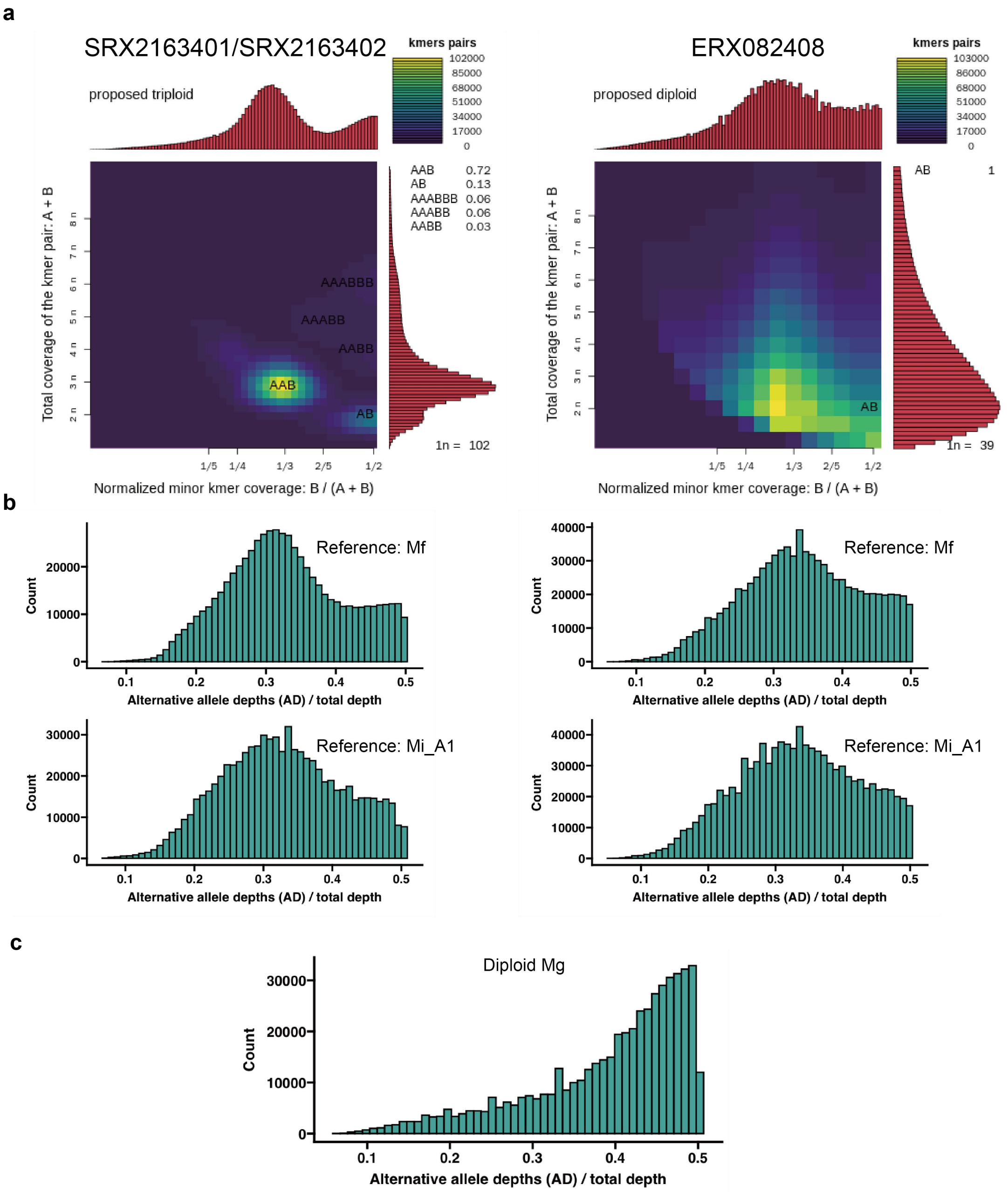

Supplementary Figure 39 Evidence for ploidy of *M. floridensis.* (**a**) Smudgeplots of two *M. floridensis* accessions showing the ploidy estimation. (**b**) Alternative allele depths for heterozygous SNPs in *M. floridensis* data. (**c**) Alternative allele depths for heterozygous SNPs in *M. graminicola* data. Peaks of alternative allele ratio are 0.33 in *M. floridensis* but 0.5 in *M. graminicola* (diploid), indicating that *M. floridensis* are triploid species.

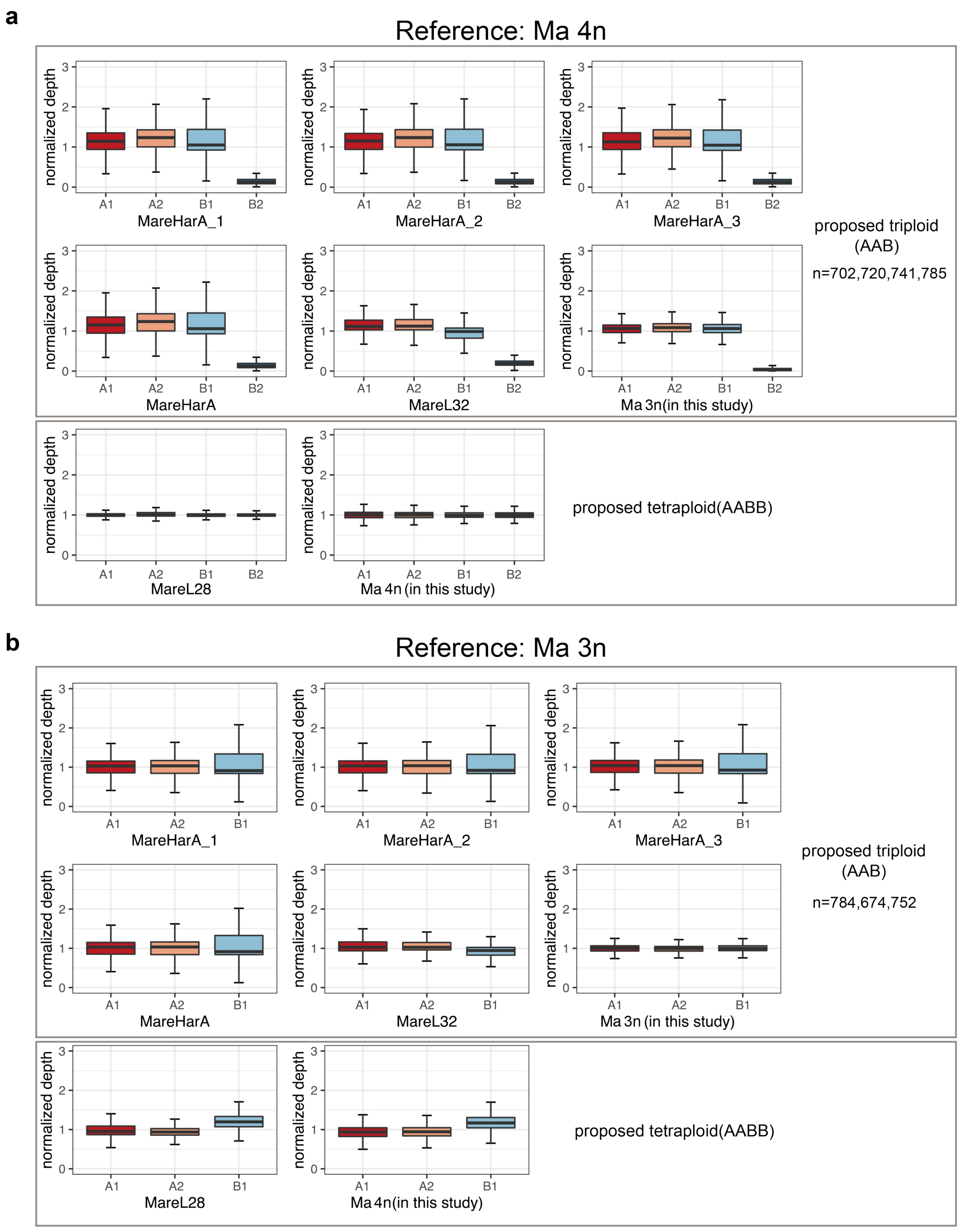

Supplementary Figure 40 Two lineages in current defined species *M. arenaria*. (**a**) Boxplot of normalized reads depth for alignment obtained from Ma accessions mapped into Ma 4n genome (assembly v2). (**b**) Boxplot of normalized reads depth for alignment obtained from Ma accessions mapped into Ma 3n genome (assembly v2). This reveals that some Ma accessions are proposed triploid with lacking B_2 genome. In box plots, the central line represents the median, the box represents the 25% and 75% percentiles, and the whiskers represent 1.5 times the interquartile range beyond the box. Source data are provided as a Source Data file.

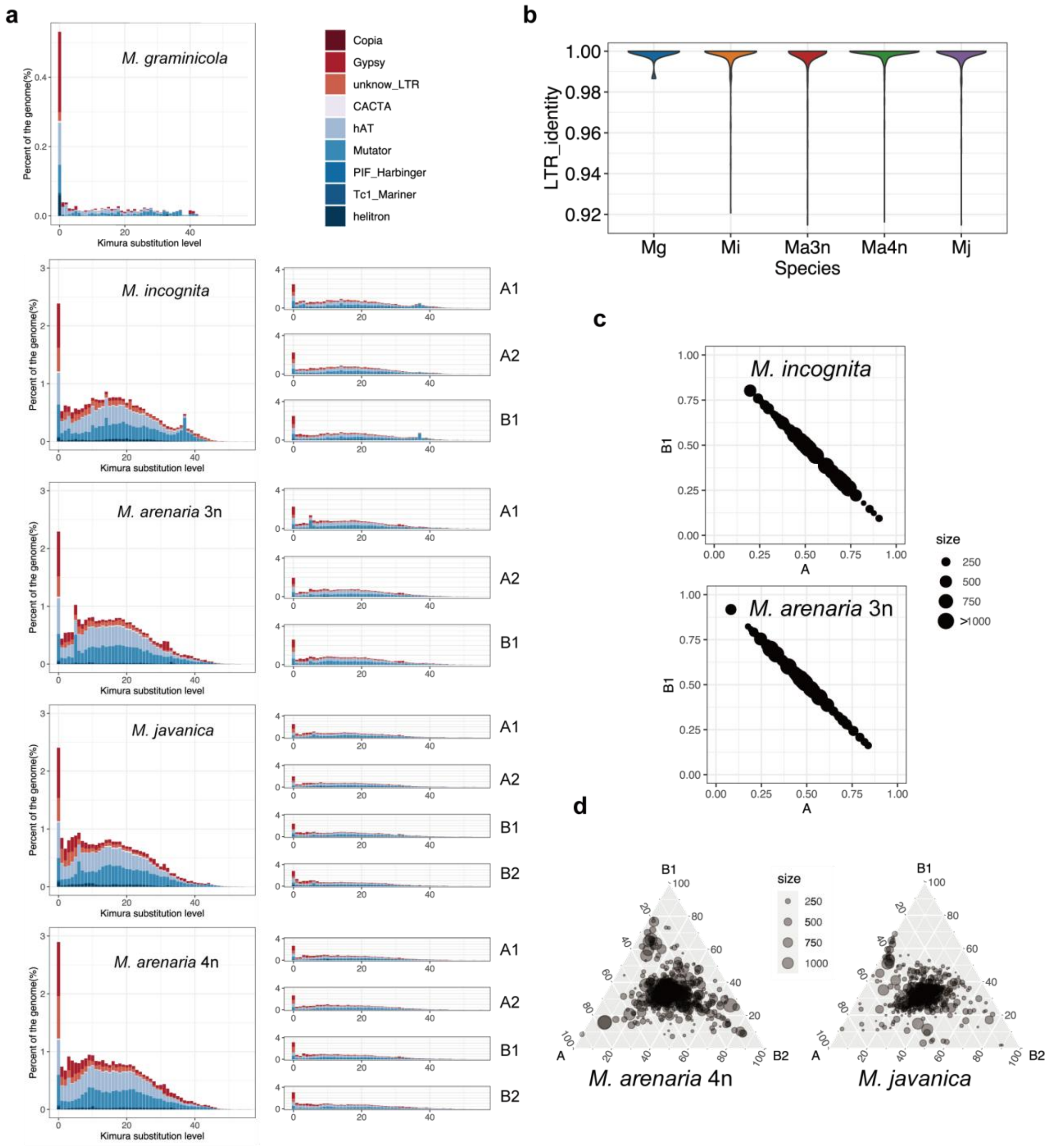

Supplementary Figure 41 TE dynamic in polyploid species. (**a**) Divergence of repetitive sequences in five genomes. Kimura substitution levels were calculated for all TE copies found within each family to estimate the age of TE insertions. In the distribution, the closer the peak position is to 0, the younger the corresponding TE is. (**b**) LTR identity of intact LTR retrotransposons in five genomes. (**c**) Relative abundance of each TE family in triploid species. (**d**) Relative abundance of each TE family in tetraploid species. The abundance in the A genome is the average of A_1 and A_2 genome. Only TE family with more than 50 copy are shown. Source data are provided as a Source Data file.

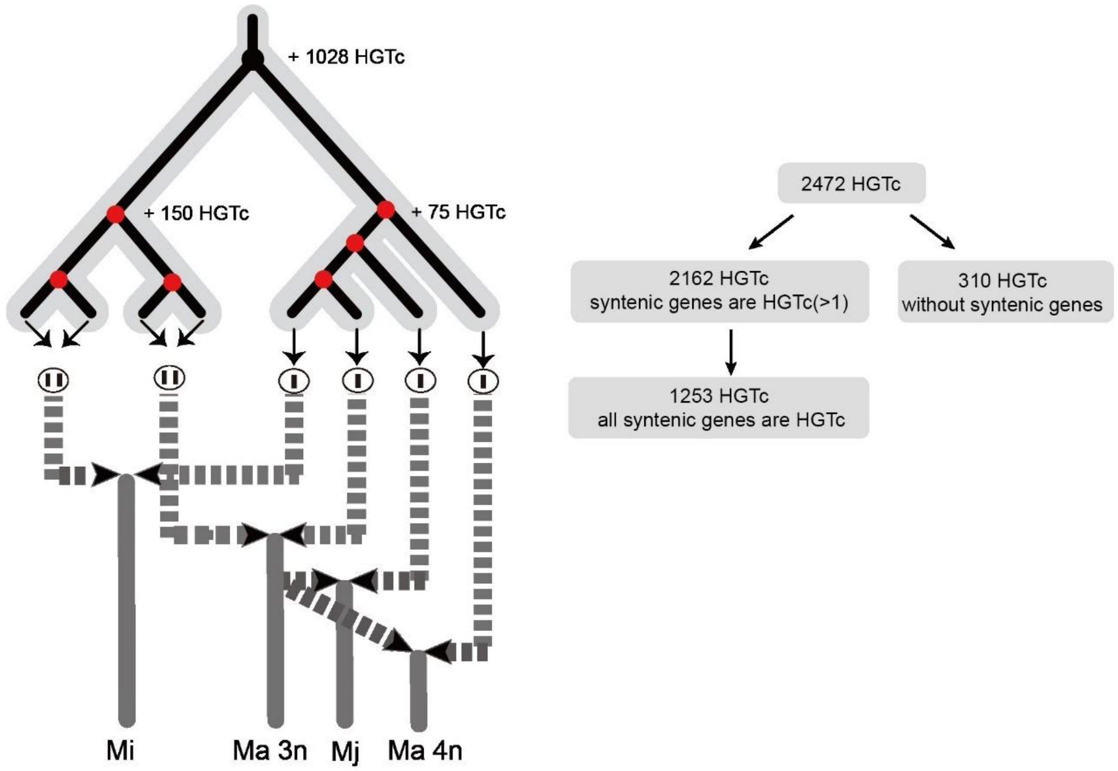

Supplementary Figure 42 Synteny analysis in HGTc (HGT candidates). Only HGTc where all syntenic genes are HGTc is used for subsequent analysis. For each HGTc, the gene is considered ancestrally acquired, when the gene and its syntenic genes are present in both the A and B subgenome.

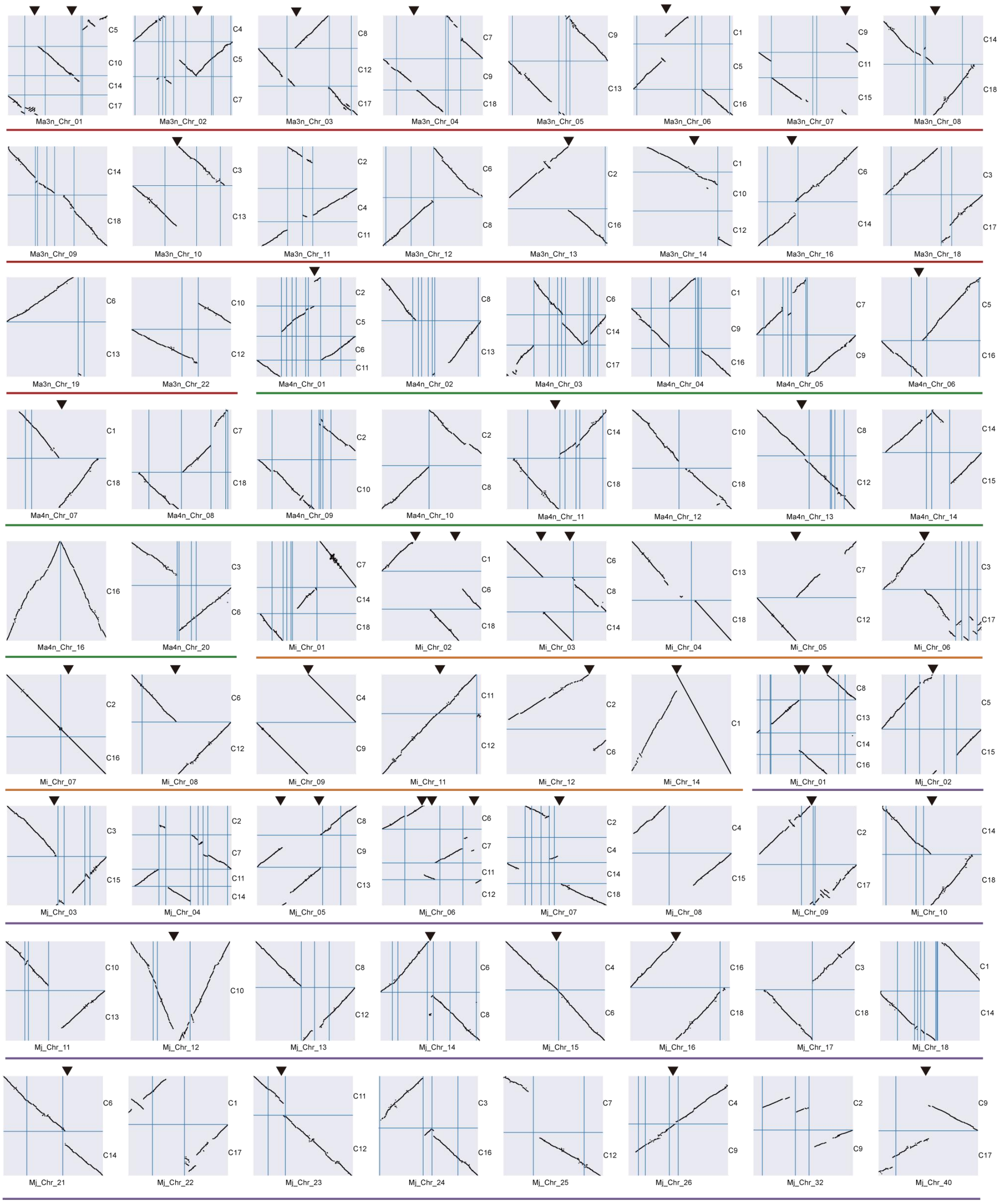

Supplementary Figure 43 Chromosomal fusions in polyploid species. Dot plots show the genomic synteny between fused chromosome and the corresponding ancestral chromosome. The vertical lines represent gaps between contigs (For Mi, BioNano improved contigs are used). The black triangles indicate that fusions are present at the contig level.

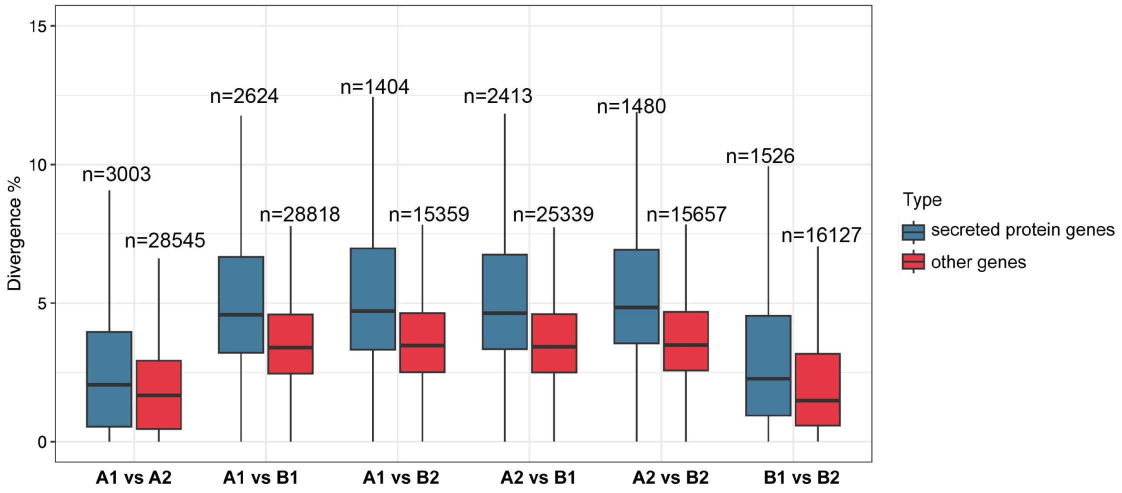

Supplementary Figure 44 Sequence divergence among secreted protein genes and other genes (total genes without secreted protein genes). Boxplot shows the nucleotide divergence among subgenomes from difference ancestor for secreted protein genes and other genes. Sequence divergence are calculated by this type of gene between the two subgenomes in four species. Source data are provided as a Source Data file.
