# Supplementary material for: Unzipped chromosome-level genomes reveal allopolyploid nematode origin pattern as unreduced gamete hybridization
Source: Nat Commun. 2023 Nov 7;14:7156. doi: 10.1038/s41467-023-42700-w (PMC10630426; doi:10.1038/s41467-023-42700-w)
Supplement: Supplementary file 3 — Description of Additional Supplementary Files [file 41467_2023_42700_MOESM3_ESM.pdf]

Description of Additional Supplementary Files

Supplementary Data 1: Summary of sequencing data for assembly v1

Supplementary Data 2: Summary of data set for mitochondrial phylogenetic analysis

Supplementary Data 3: Genome size estimation for five RKNs

Supplementary Data 4: Summary of chromosome-level assembly for *M. graminicola* (assembly v1)

Supplementary Data 5: Statistics of assembly for polyploid species in assembly v1

Supplementary Data 6: BUSCO statistic for assembly in this study and other Meloidogyne species genome (genome model)

Supplementary Data 7: Summary of repetitive sequences for five RKN genomes (assembly v1)

Supplementary Data 8: Statistics of gene annotation for five RKN genomes (assembly v1)

Supplementary Data 9: BUSCO statistic for assembly in this study and other Meloidogyne species genome (proteome model)

Supplementary Data 10: Subgenome information for assembly v1

Supplementary Data 11: Telomere-associated genes surveyed in this study

Supplementary Data 12: Consensus sequence of telomeric repeats in M. graminicola (Mg-Tel)

Supplementary Data 13: Summary of BioNano data for *M. incognita*

Supplementary Data 14: Summary of Nanopore data and assembly

Supplementary Data 15: Statistics of chromosome-level genome for polyploid RKNs (assembly v2)

Supplementary Data 16: Chromosome size of assembly v2

Supplementary Data 17: Summary of chromosome-level assembly for *M. graminicola* (T2T)

Supplementary Data 18: Information of HGT candidates

Supplementary Data 19: Splitting of Mi chromosomes based on genomic synteny between Mi (v2) and Mg(T2T)

Supplementary Data 20: Splitting of Ma 3n chromosomes based on genomic synteny between those with Mi B1 subgenome

Supplementary Data 21: Splitting of Ma 4n chromosomes based on genomic synteny between those with Mi B1 subgenome

Supplementary Data 22: Splitting of Mj chromosomes based on genomic synteny between those with Mi B1 subgenome.

Supplementary Data 23: Subgenome information for polyploid RKNs (Assembly v2)

Supplementary Data 24: Sequence divergence (coding gene) between 14 subgenome in nucleotide level and amino acid level

Supplementary Data 25: Chromosomal fusion events in polyploid RKNs

Supplementary Data 26: Definition of homoeolog expression bias categories for polyploid RKNs

Supplementary Data 27: Summary of gene expression pattern among five development stage for polyploid RKNs

Supplementary Data 28: Detailed information of gene expression pattern.
