# Supplementary material for: Unzipped chromosome-level genomes reveal allopolyploid nematode origin pattern as unreduced gamete hybridization
Source: Nat Commun. 2023 Nov 7;14:7156. doi: 10.1038/s41467-023-42700-w (PMC10630426; doi:10.1038/s41467-023-42700-w)
Supplement: Supplementary file 6 — Reporting Summary [file 41467_2023_42700_MOESM6_ESM.pdf]

nature portfolio

Corresponding author(s): Donghai Peng, Jinshui Zheng, and Ming Sun

Last updated by author(s): Aug 6, 2023

Reporting Summary

Nature Portfolio wishes to improve the reproducibility of the work that we publish. This form provides structure for consistency and transparency in reporting. For further information on Nature Portfolio policies, see our Editorial Policies and the Editorial Policy Checklist.

Statistics

For all statistical analyses, confirm that the following items are present in the figure legend, table legend, main text, or Methods section.

n/a	Confirmed	
☐	☒	The exact sample size (*n*) for each experimental group/condition, given as a discrete number and unit of measurement
☒	☐	A statement on whether measurements were taken from distinct samples or whether the same sample was measured repeatedly
☐	☒	The statistical test(s) used AND whether they are one- or two-sided<br>*Only common tests should be described solely by name; describe more complex techniques in the Methods section.*
☒	☐	A description of all covariates tested
☒	☐	A description of any assumptions or corrections, such as tests of normality and adjustment for multiple comparisons
☐	☒	A full description of the statistical parameters including central tendency (e.g. means) or other basic estimates (e.g. regression coefficient) AND variation (e.g. standard deviation) or associated estimates of uncertainty (e.g. confidence intervals)
☐	☒	For null hypothesis testing, the test statistic (e.g. *F*, *t*, *r*) with confidence intervals, effect sizes, degrees of freedom and *P* value noted<br>*Give P values as exact values whenever suitable.*
☒	☐	For Bayesian analysis, information on the choice of priors and Markov chain Monte Carlo settings
☒	☐	For hierarchical and complex designs, identification of the appropriate level for tests and full reporting of outcomes
☒	☐	Estimates of effect sizes (e.g. Cohen's *d*, Pearson's *r*), indicating how they were calculated

Our web collection on statistics for biologists contains articles on many of the points above.

Software and code

Policy information about availability of computer code

Data collection	no software was used to collect data
Data analysis	Genome assembly: SMRTdenovo, Canu(v1.9), Canu(v2.1), Nextpolish(v1.1.0), Solve(v3.4.1), Juicer(v1.5.7), 3D-DNA(180922), Juicebox(v1.13.01), MapOptics(v2.0).<br>Genome completness assessment: CEGMA(v2.5),BWA(0.7.17), bedtools(v2.30.0)<br>Genome annotation: HISAT(v2.2.0), Braker(v2.1.5), EDTA(v2.0.0), RepeatMasker(v4.0.9), Trinity(v2.9.0), IsoSeq3, CD-HIT(v4.8.1), PASA(v2.4.1), StringTie(2.1.1), TransDecoder(v5.5.0), AUGUSTUS(3.3.3), GeneMark-ES(v4.69), EVidenceModeler(v1.1.1), TMHMM(2.0), SignalP(5.0), eggNOG-mapper(v2), HMMER(3.3).<br>Synteny analysis: MCScanX, jcvi.<br>Hi-C matrix construction: HiC-pro(v3.1.0).<br>Phylogenetic analysis: MAFFT(v7.471), GBLOCKS(0.91b), IQ-TREE(v2.0.3), KaKs_Calculator(2.0), SAMtools(v1.7), FreeBayes(v0.9.21).<br>Telomere analysis: BLSATP, minimap2(2.17), MUMmer(4.0.0).<br>Gene expression analysis: Kallisto(v0.48.0).<br>Visualization: iTol, circos.<br>R packages: pheatmap, Rideogram, ggtree.<br><br>All the scripts and pipelines used in this study are openly available on github (https://github.com/xiecs-BMB/MIG-genome).

For manuscripts utilizing custom algorithms or software that are central to the research but not yet described in published literature, software must be made available to editors and reviewers. We strongly encourage code deposition in a community repository (e.g. GitHub). See the Nature Portfolio guidelines for submitting code & software for further information.

Data

Policy information about availability of data

All manuscripts must include a data availability statement. This statement should provide the following information, where applicable:

- Accession codes, unique identifiers, or web links for publicly available datasets
- A description of any restrictions on data availability
- For clinical datasets or third party data, please ensure that the statement adheres to our policy

All sequencing data have been deposited into the National Center for Biotechnology Information (NCBI) Sequence Read Archive (SRA). The PacBio, Illumina, and Hi-C data generated in this study have been desposited in SRA database under accession code PRJNA784524; The RNA-seq data generated in this study have been desposited in SRA database under accession code PRJNA786696; The BioNano data generated in this study have been desposited in SRA database under accession code PRJNA787730; The Nanopore data generated in this study have been desposited in SRA database under accession code PRJNA788579; The ISO-seq data of M. incognita generated in this study have been desposited in SRA database under accession code PRJNA787737; The assembly genome and annotation files generated in this study have been desposited in our laboratory website; The accession codes of data set for mitochondrial phylogenetic analysis are in Supplementary Table 2; The data set of genome and Illumina reads downloaded for telomere structure and repeat count analysis are under accession codes GCA_904067135.1, GCA_022814885.1, GCA_000724045.1, GCA_900079975.1, GCA_019095935.1, GCA_004148225.1, GCA_015183035.1, GCA_018905775.1, GCA_000172435.1, GCA_003693605.1, GCA_000751915.1, GCA_903994135.1, GCA_902706615.1, SRR16969916, DRR067231, ERR123957, ERR123958, SRR1800546, SRR15101032; The data set downloaded for histone modification analysis of M. incognita is under accession code PRJNA725801.

Research involving human participants, their data, or biological material

Policy information about studies with human participants or human data. See also policy information about sex, gender (identity/presentation), and sexual orientation and race, ethnicity and racism.

Reporting on sex and gender	N/A
Reporting on race, ethnicity, or other socially relevant groupings	N/A
Population characteristics	N/A
Recruitment	N/A
Ethics oversight	N/A

Note that full information on the approval of the study protocol must also be provided in the manuscript.

Field-specific reporting

Please select the one below that is the best fit for your research. If you are not sure, read the appropriate sections before making your selection.

☒ Life sciences ☐ Behavioural & social sciences ☐ Ecological, evolutionary & environmental sciences

For a reference copy of the document with all sections, see nature.com/documents/nr-reporting-summary-flat.pdf

Life sciences study design

All studies must disclose on these points even when the disclosure is negative.

Sample size	No sample size calculations were performed. Sample sizes were determined by: a) technical tractability - for the extremely challenging sample collection for cross-kingdom transcriptomics, three repetitions could be reasonably achieved and because statistical difference calculations require at least two repetitions and take into account sample size and variance. Therefore, all RNA-seq in this study were performed in three biological replicates; and b) reference the previous experience working with plant-parasitic nematode genome research (Siddique S, Radakovic ZS, Hiltl C, et al. The genome and lifestage-specific transcriptomes of a plant-parasitic nematode and its host reveal susceptibility genes involved in trans-kingdom synthesis of vitamin B5. Nat Commun. 2022;13(1):6190.).
Data exclusions	No data exclusion in our analysis.
Replication	All attempts at replication were successful.
Randomization	Randomization sample allocation were used for gene expression by random assigning the selected individuals to different groups.
Blinding	Blind experiments are not used in this study. The experiments involved in this study include RCR, Hi-C library construction, RNA-seq library construction, DNA long and short read library construction, DNA telomeric FISH experiments, etc., and do not involve the experimental group and the control group. The experimental group and the control group are also not involved in the data analysis process. Therefore, the results will not be affected by subjective factors, so no single-blind experiment or double-blind experiment was carried out.

Reporting for specific materials, systems and methods

We require information from authors about some types of materials, experimental systems and methods used in many studies. Here, indicate whether each material, system or method listed is relevant to your study. If you are not sure if a list item applies to your research, read the appropriate section before selecting a response.

Materials & experimental systems

n/a	Involved in the study
☒	☐ Antibodies
☒	☐ Eukaryotic cell lines
☒	☐ Palaeontology and archaeology
☐	☒ Animals and other organisms
☒	☐ Clinical data
☒	☐ Dual use research of concern
☒	☐ Plants

Methods

n/a	Involved in the study
☒	☐ ChIP-seq
☒	☐ Flow cytometry
☒	☐ MRI-based neuroimaging

Animals and other research organisms

Policy information about studies involving animals; ARRIVE guidelines recommended for reporting animal research, and Sex and Gender in Research

Laboratory animals	We did not use laboratory animals.
Wild animals	Polyploid MIG isolates (M. incognita, M. javanica, M. arenaria 4n) were collected from Hubei, Fujian and Liaoning province of China. Another nematode M. arenaria 3n was collected from Yunnan province. All nematodes were collected from infected vegetables.
Reporting on sex	The sex was not considered in this study.
Field-collected samples	After field sampling, to obtain a pure single nematode lineage for DNA sequencing, we separated single egg mass from each species (Mi, Ma 4n, Mj, Ma 3n, and Mg) and inoculated it to Rutgers tomato in greenhouse at 25 °C constant temperature. After 1-4 generations of single egg mass purification, the eggs of the nematode were collected and used for constructing sequencing library.
Ethics oversight	The studies in this work have received ethical approval from the board of ethic committee, State Key Laboratory of Agricultural Microbiology, Huazhong Agricultural University, Wuhan, China.

Note that full information on the approval of the study protocol must also be provided in the manuscript.
